# Probing the nucleobase selectivity of RNA polymerases with dual-coding substrates

**DOI:** 10.1016/j.jbc.2024.107755

**Published:** 2024-09-12

**Authors:** Janne J. Mäkinen, Petja Rosenqvist, Pasi Virta, Mikko Metsä-Ketelä, Georgiy A. Belogurov

**Affiliations:** 1 Department of Life Technologies, University of Turku, Turku, Finland; 2 Department of Chemistry, University of Turku, Turku, Finland

**Keywords:** bacterial transcription, substrate specificity, nucleoside analog, enzyme kinetics, RNA polymerase, formycin, yprazofurin, ribavirin, 8-oxoguanine, 8-oxoadenine

## Abstract

Formycin A (FOR) and pyrazofurin A (PYR) are nucleoside analogs with antiviral and antitumor properties. They are known to interfere with nucleic acid metabolism, but their direct effect on transcription is less understood. We explored how RNA polymerases (RNAPs) from bacteria, mitochondria, and viruses utilize FOR, PYR, and oxidized purine nucleotides. All tested polymerases incorporated FOR in place of adenine and PYR in place of uridine. FOR also exhibited surprising dual-coding behavior, functioning as a cytosine substitute, particularly for viral RNAP. In contrast, 8-oxoadenine and 8-oxoguanine were incorporated in place of uridine in addition to their canonical Watson–Crick codings. Our data suggest that the interconversion of canonical *anti* and alternative *syn* conformers underlies dual-coding abilities of FOR and oxidized purines. Structurally distinct RNAPs displayed varying abilities to utilize *syn* conformers during transcription. By examining base pairings that led to substrate incorporation and the entire spectrum of geometrically compatible pairings, we have gained new insights into the nucleobase selection processes employed by structurally diverse RNAPs. These insights may pave the way for advancements in antiviral therapies.

Formycin A (FOR) and pyrazofurin A (PYR) are related nucleoside analogs produced by *Streptomyces* and other actinobacteria. FOR and PYR are the products of similar biosynthetic pathways (1, 2) and belong to C-nucleoside antibiotics (3). Their original functionality in actinobacteria is presumably to suppress the growth of competing organisms. These compounds have also been explored as anticancer and antiviral agents, but their clinical use has been limited due to toxicity (3, 4, 5).

FOR is similar to adenosine but contains a pyrazolopyrimidine moiety in place of imidazolopyrimidine featured by adenosine (Fig. 1*A*). PYR closely resembles ribavirin (RIB), a synthetic nucleoside analog that was originally developed as an antiviral drug (Fig. 1*A*) (6). Both PYR and RIB can be viewed as incomplete purine analogs featuring a 5-membered ring and a carboxamide moiety. The 5-membered ring of the nucleobase is pyrazole in PYR and triazole in RIB. In addition, the PYR nucleobase features a hydroxyl group in ortho position relative to the carboxamide moiety.

**Figure 1:**
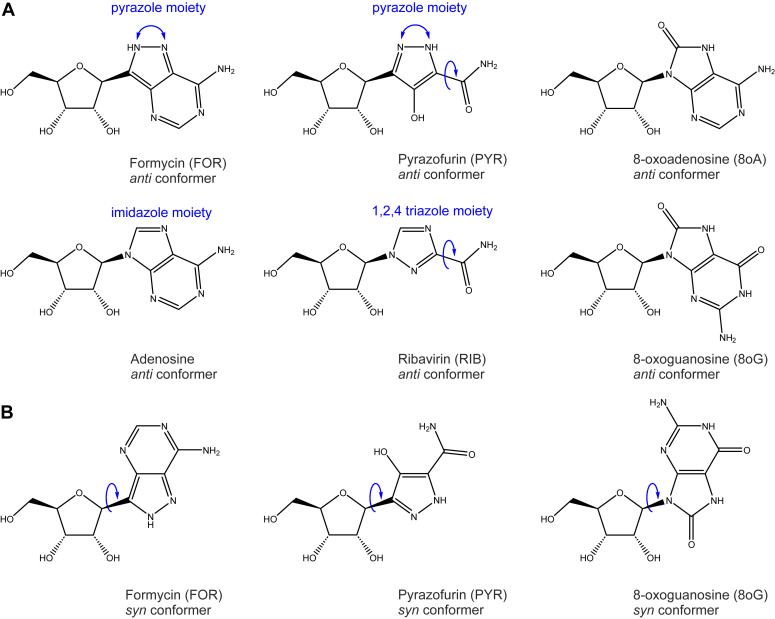
**Nucleoside analogs employed to study the nucleobase selectivity of diverse RNAPs.***A,* FOR closely resembles adenosine but contains pyrazole moiety in place of the imidazole moiety of adenosine. PYR resembles FOR but contains an incomplete six-membered ring. PYR also resembles ribavirin (RIB): both compounds feature a carboxamide moiety attached to the five-membered aromatic ring that is pyrazole in PYR and triazole in RIB. The carboxamide moiety can rotate 180° (*curved blue arrows*), enabling PYR and RIB to resemble either adenine or guanine. Pyrazole moieties of FOR and PYR can equilibrate between two tautomers (*curved blue arrows*). 8oA and 8oG feature a keto group at position corresponding to C8 of adenosine and guanine and a polar hydrogen at a nitrogen corresponding to N7 of adenosine and guanine. *B*, FOR, PYR, 8oA, and 8oG have the increased propensity to adopt a so-called *syn* configuration by rotating around the glycosidic bond (*curved blue arrows*). 8oA, 8-oxoadenine; 8oG, 8-oxoguanine; FOR, formycin A; PYR, pyrazofurin A; RNAP, RNA polymerase.

FOR and PYR differ from adenosine and RIB by featuring C-C glycosidic bonds and are thus resistant to the action of cellular glycosylases. The 1.55 Å C-C bond is also slightly longer than the 1.47 Å C-N bond, lowering the energy barrier for the nucleobase rotation around the glycosidic bond and facilitating the reversible interconversion between *anti* and *syn* conformers (Fig. 1*B*) (7, 8, 9). In crystals, FOR and PYR adopt *syn* (CCDC IDs FOMHBH10, PYRZOM01), intermediate (CCDC ID FORMYC, PDB IDs 1K9S, 1T8S, 3UT6), and *anti*-like conformations (PDB IDs 3MI2, 3SEC). The second major difference is the presence of a polar hydrogen at one of the nitrogens of the pyrazole moiety. Both FOR and PYR exist as a mixture of two readily interconverting tautomers (10). The tautomer with the polar hydrogen at the nitrogen adjacent to the glycosidic bond (Fig. 1*A*) may become the predominant form upon FOR binding to the enzyme active sites (11).

FOR and PYR are thought to interfere with the metabolism of nucleic acids, though the exact metabolic steps affected by these compounds and the mechanism of inhibition are not fully understood (12). FOR has been reported to target purine nucleoside phosphorylase (13, 14), whereas PYR targets orotidine-5′-monophosphate decarboxylase (15, 16, 17). FOR, PYR, and RIB are also recognized by nucleoside kinases when inside the cell and undergo series of phosphorylation reactions, ultimately converting into the triphosphate forms (6, 17, 18) that can serve as substrates for nucleic acid polymerases (14, 19, 20). FOR is *a priori* expected to function as an adenine analog: both compounds feature identical amino substituted pyrimidine rings that can mediate base pairing with uridine or thymine. The pyrazole ring of FOR is not expected to affect base pairing in the canonical *anti* conformation of the nucleobase but may affect stacking with adjacent nucleobases during and after incorporation into RNA as we previously proposed for oxazinomycin (21). In contrast, the carboxamide group of PYR and RIB can rotate 180° potentially enabling these compounds to mimic either adenine or guanine depending on the orientation of the carboxamide group (20).

Nucleobases that can form base pairs with more than one nucleobase in the active sites of nucleic acid polymerases are frequently described as dual coders. Some of the best-known dual coders are oxidized derivatives of nucleosides, 8-oxoguanine (8oG) and 8-oxoadenine (8oA) (Fig. 1). 8oG pairs with cytosine and adenine when in *anti* and *syn* configuration, respectively (22, 23). 8oA predominantly pairs with uridine and guanine when in *anti* and *syn* configuration, respectively (24, 25). In general, the dual coding potential may result from the rotation of a moiety as in RIB (20), rotation of the entire nucleobase around the glycosidic bond as in 8oG, or reversible interconversion of tautomeric forms as in N4-hydroxycytidine (26, 27). Deoxyribonucleoside versions of dual coders are potent mutagens in DNA genomes of cellular life forms (28, 29, 30). In contrast, ribonucleoside versions of dual coders can cause translational errors and have mutagenic effects in genomes of RNA viruses (20, 26, 31).

In this study, we investigated utilization of triphosphorylated forms of FOR, PYR, and structurally related nucleoside analogs RIB, 8oA, and 8oG by three major types of RNA polymerases (RNAPs). *Escherichia coli* enzyme (Ec-DdRP) served as a representative of DNA-dependent RNAPs (DdRPs) that belong to the two-barrel structural family (two double-psi-β-barrel domains constitute a part of the active site) (32, 33, 34, 35). These are commonly referred to as multisubunit RNAPs, but we found it advantageous to call them two-barrel DdRPs in this study. Two-barrel DdRPs are responsible for transcription of genomes in bacteria, archaea, and eukaryotic nuclei (36). Human mitochondrial RNAP (Mt-DdRP) was employed as a DdRP representative of the right-hand structural family of nucleic acid polymerases (the catalytic module resembles the right hand) (37, 38). Right-hand DdRPs transcribe mitochondrial genomes across all eukaryotic organisms (39, 40) and certain segments of chloroplast genomes in higher plants (41). Finally, RNAP from coxsackievirus (Cv-RdRP) served as an RNA-dependent representative (RdRP) of the right-hand structural family of nucleic acid polymerases (42, 43, 44). Right-hand RdRPs transcribe and replicate genomes of RNA viruses such as poliovirus (45), hepatitis C virus (46), coxsackievirus (47), and coronaviruses (48).

Despite considerable structural and evolutionary divergence, all three major types of RNAPs feature a principally similar geometry of the active site: (*i*) the substrate NTP is recruited by forming a Watson–Crick base pair with the acceptor nucleotide in the template strand; (*ii*) the ribose moiety of NTP is recognized and bound in a dedicated pocket; and (*iii*) the nucleotide addition occurs at the primer-template junction where the acceptor nucleotide, the primer-terminal RNA nucleotide and the substrate NTP are all arranged according to the A-form helical geometry. By employing three diverse RNAPs, we aimed to both delineate the base pairing capabilities of nucleoside analogs and pinpoint the differences in the nucleobase selection mechanism employed by diverse RNAPs. Our results reveal several unexpected codings of nucleoside analogs that are likely mediated by *syn* conformers of nucleoside triphosphates. We further observed differential utilization of these codings by different RNAPs in our set. By examining the base pairings responsible for substrate incorporation and the entire spectrum of base pairings with potential geometric compatibility, we have gained new insights into the nucleobase selection processes employed by three major types of RNAPs. These insights may pave the way for future advancements in antiviral therapies.

## Results

### The setup of single-nucleotide addition experiments

We synthesized triphosphates of FOR and PYR using the previously established method (21, 49) and acquired triphosphorylated RIB, eight-8oA and 8oG from commercial sources. We then investigated the ability of Ec-DdRP, Mt-DdRP, and Cv-RdRP to utilize the triphosphorylated nucleoside analogs in place of natural substrates ATP, CTP, GTP, and UTP.

**Figure 2:**
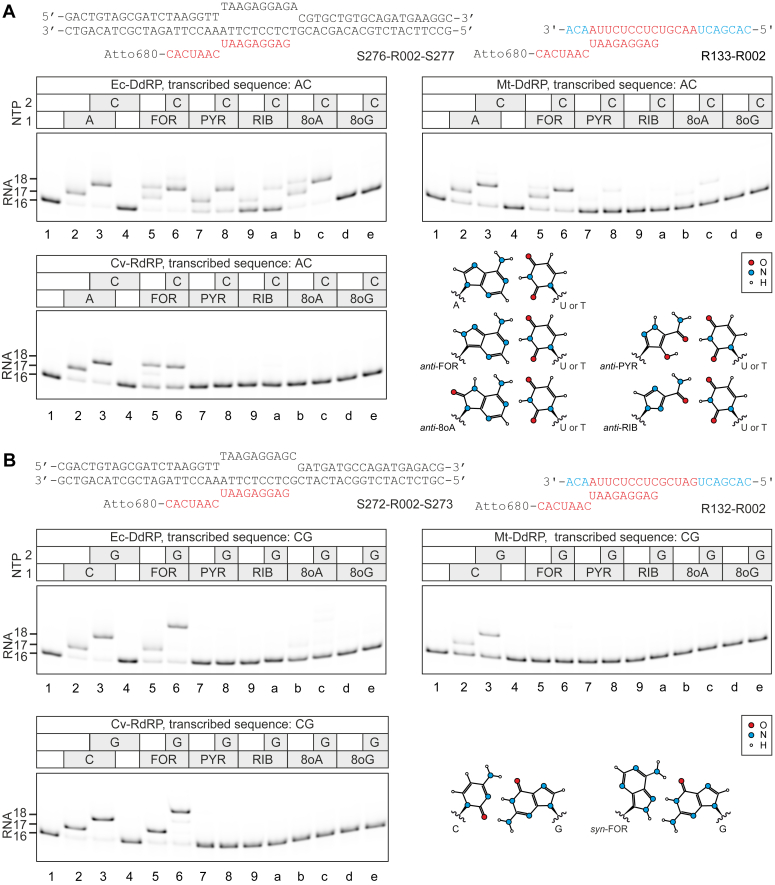
**Incorporation of nucleoside analogs into RNA in place of adenine and cytidine by Ec-DdRP, Mt-DdRP, and Cv-RdRP.** Assembled ECs were supplemented with 10 μM NTPs, 100 μM nucleoside analogs, and incubated for 1 min at 25 °C. *A*, incorporation in place of adenine. *B*, incorporation in place of cytidine. Schematics of nucleic acid scaffolds are shown above gel panels. DNA, RNA, and 2′OMe nucleotides are colored *black*, *red*, and *cyan*, respectively. Quantification is presented in the Supplementary Data File. 2′-OMe, 2′-O-methylated; DdRP, DNA-dependent RNAP; EC, elongation complex; Mt-DdRP, mitochondrial RNAP; Cv-RdRP, RNAP from coxsackievirus.

**Figure 3:**
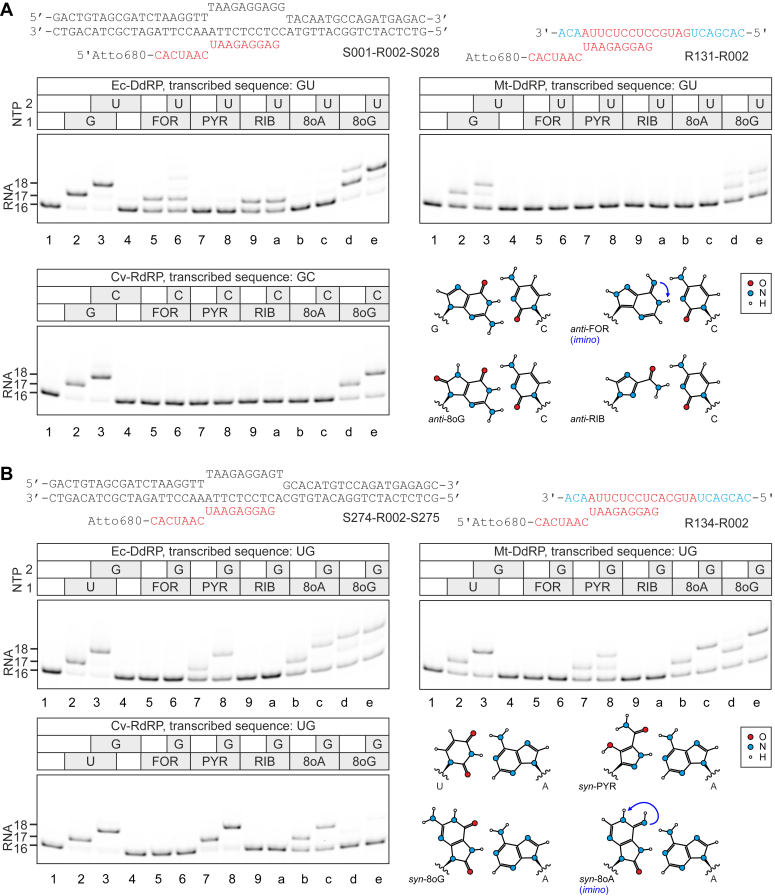
**Incorporation of nucleoside analogs into RNA in place of guanine and uridine by Ec-DdRP, Mt-DdRP, and Cv-RdRP.** Assembled ECs were supplemented with 10 μM NTPs, 100 μM nucleoside analogs, and incubated for 1 min at 25 °C. *A*, incorporation in place of guanine. *B*, incorporation in place of uridine. Schematics of nucleic acid scaffolds are shown above gel panels. DNA, RNA and 2′OMe nucleotides are colored *black*, *red*, and *cyan*, respectively. Quantification is presented in the Supplementary Data File. 2′-OMe, 2′-O-methylated; Cv-RdRP, RNAP from coxsackievirus; DdRP, DNA-dependent RNAP; EC, elongation complex; Mt-DdRP, mitochondrial RNAP.

Transcription elongation complexes (ECs) were assembled on synthetic nucleic acid scaffolds. The scaffolds for DdRPs contained a fully complementary transcription bubble flanked by 20-nt-long DNA duplexes upstream and downstream (Figs. 2 and 3). The annealing region of a 16-nt RNA primer was nine nucleotides. The RNA primer was 5′ labeled with the infrared fluorophore ATTO680 to monitor RNA extension by denaturing PAGE. To assemble RdRP ECs, the same 16-nt RNA primer was annealed to the 24-nt-long RNA template that contained 2′-O-methylated (2′-OMe) nucleotides at the 3′ and 5′ ends to increase its stability against degradation by exonucleases. The resulting scaffold contained a 9-base pair RNA:RNA duplex, 3 2′-OMe nucleotides upstream, 5 nt of single-stranded template RNA downstream of the primer-template junction followed by 7 2′-OMe nucleotides. Four RNA:DNA and four RNA:RNA scaffolds were designed so that the first incorporated nucleotide was AMP, CMP, GMP, or UMP. When the first incorporating nucleotide was purine, the second was pyrimidine and *vice versa* to minimize readthrough due to misincorporation of canonical nucleoside monophosphates (NMPs).

We then walked RNAPs on these four scaffolds by one or two positions utilizing either two canonical substrates or a nucleoside analog and a canonical substrate (Figure 2, Figure 3). These experiments revealed whether the nucleoside analog can be incorporated in place of a canonical substrate and whether the resulting EC can incorporate the next nucleotide. Before introducing the results of the walking experiments it is imperative to consider two aspects of the experimental setup and data interpretation.

First, RNAPs are highly selective toward the cognate NTPs (NTPs that form canonical Watson–Crick base pairs with the acceptor nucleotide in the template DNA or RNA) but will also incorporate non-cognate NMPs if incubated with noncognate NTPs for sufficiently long time. In our experiments, the reaction time (1 min) and nucleoside analog concentrations (100 μM) were chosen to permit detection of incorporation events with about ∼1000-fold slower rate than that for canonical NTPs (50, 51, 52). Under such conditions, most misincorporation events involving canonical substrates were not observed, except for the uridine measurably incorporating in place of cytidine and adenine in place of guanine (Fig. S1). In other words, our walking experiments were performed at the threshold conditions where some misincorporation of canonical NMPs become noticeable but were still relatively inefficient. In this study, we describe a nucleoside analog as recognized by RNAP in place of a canonical cognate nucleotide when its relative incorporation efficiency exceeds 25% under our standard assay conditions (100 μM substrate, 1 min, 25 °C). This threshold represents the relative efficiency of most efficient misincorporations of canonical nucleotides in our assay.

Second, we found it imperative to model tentative geometries of base pairings suggested by our data and present such analysis alongside the experimental observations. To do so, we postulated that a productive base pairing (base pairing that leads to substrate incorporation) must (*i*) follow Watson–Crick geometry, and (*ii*) feature similar distances between the nucleo-sugars to those observed in canonical A-T, A-U, and G-C base pairs. These postulates have been largely proven for high-fidelity DNA polymerases (22, 53, 54, 55, 56) and it is conceivable that they hold also for RNAPs. To graphically implement these postulates, we started with 2D projections of the canonical pairs between nucleobases including glycosidic bonds. To draw the tentative geometries of base pairs formed by nucleoside analogs, we replaced the canonical nucleobases with their analogs while maintaining the distance between the glycosidic bonds and, where necessary, introducing only minimal changes to the angle between the glycosidic bonds (Fig. S2). We explored both canonical *anti* and less frequently encountered *syn* orientations of the nucleobases. For *syn*-*anti* pairs, we rotated the glycosidic bonds of the substrate nucleobase by 10°, which is within the normal variations of the glycosidic bond angle in canonical nucleotides. Finally, we acknowledge the possibility of incorporations mediated exclusively by base stacking to the primer-terminal nucleobase (*e. g.,* incorporations against an abasic site) and specialized geometries observed in the active sites of translesion DNA polymerases, where wobble pairing may result in nucleotide incorporation (57). However, we did not need to invoke stacking-only scenarios to explain our data and considered that wobble pairings are unlikely to be productive in the active sites of RNAPs.

### Utilization of nucleoside analogs in single-nucleotide addition experiments

FOR was incorporated in place of adenine by all RNAPs in our set as expected from the coding capacity of the *anti* conformer (Fig. 2*A* lanes 5–6). Unexpectedly, FOR was also recognized as cytidine by Ec-DdRP and Cv-RdRP but not Mt-DdRP (Fig. 2*B* lanes 5–6). C-coding of FOR was also evident from a partial readthrough by two positions during transcription of the AC sequence in the presence of FOR (Fig. 2*A* lane 5). The readthrough efficiency declined in the order Cv-RdRP > Ec-DdRP > > Mt-DdRP. We reasoned that pairing with guanine is most likely mediated by the *syn* conformer of FOR (Fig. 2*B*) because *syn*-purine pairing with *anti*-purine is arguably the only possible geometry that allows for Watson–Crick-like pairing between two purines while preserving the internucleo-sugar distance characteristic for canonical purine-pyrimidine base pairs (22). FOR was also efficiently incorporated into RNA against inosine in the template DNA by Ec-DdRP (Fig. S3 lanes 7–8), indicating that exocyclic amino group of guanine is not essential for C-coding of FOR. At the same time, adenine was not incorporated against inosine (Fig. S3 lanes 5–6), suggesting that the ability of FOR to code for cytidine is primarily attributable to the increased propensity to adopt *syn* configuration.

FOR was also incorporated in place of guanine by Ec-DdRP forming an EC that failed to add the next nucleotide within the timeframe of the experiment (Fig. 3*A* lanes 5–6). Notably, a similar phenomenon was observed for ATP (Fig. S1*D* lanes 5–6). We can conceivably explain these incorporations only by invoking rare *imino* tautomers (Figs. 3*A* and S1*F*).

PYR was incorporated in place of adenine by Ec-DdRP as expected from the coding capacity of the *anti* conformer (Fig. 2*A* lanes 7–8). However, Mt-DdRP incorporated PYR in place of adenine very inefficiently (Fig. 2*A* lanes 7–8) and Cv-RdRP not at all (Fig. 2*A* lanes 7–8). At the same time, the G-coding of the *anti*-PYR corresponding to the alternative orientation of the carboxamide moiety was not observed for any RNAP in our set (Fig. 3*A* lanes 7–8). Unexpectedly, all RNAPs in our set incorporated PYR in place of uridine (Fig. 3*B* lanes 7–8). We reasoned that PYR pairing with adenine is most likely mediated by the *syn*-PYR similarly to what we proposed for FOR. Noteworthy, the hydrogen bonding of *syn*-FOR with guanine (Fig. 2*B*) and *syn*-PYR with adenine (Fig. 3*B*) both involve atoms of the pyrazole moiety but are mediated by different tautomers: *syn*-FOR accepts N1H of guanine, whereas *syn*-PYR donates hydrogen to N1 of adenine.

RIB was incorporated by Ec-DdRP in place of adenine (Fig. 2*A* lanes 9-a) and to some extent guanine (Fig. 3*A* lanes 9-a) as expected from the coding capacities of conformers with the opposite orientation of the carboxamide moiety. However, the latter EC was poorly extendable by the next nucleotide. RIB was not incorporated into RNA by right-hand RNAPs under our standard assay conditions, despite being documented to serve as a substrate for polioviral RdRP and display a dual-coding potential (20). The failure of RIB to incorporate is attributable to the relatively short reaction time that we chose to distinguish additional codings of nucleoside analogs from canonical misincorporation events. In contrast, at 1 mM, RIB was incorporated by Cv-RdRP in place of GMP and AMP on a timescale of minutes (Fig. S4), consistent with previous reports on polioviral RdRP (20). Notably, Ferrer-Orta *et al.* observed a nonincorporated RIB triphosphate in the active site of the foot-and-mouth disease virus RdRP under conditions that resulted in incorporation of other substrates such as ATP and 5-fluorouridine triphosphate (58).

8oA was incorporated in place of adenine by Ec-DdRP as expected from the coding capacity of the *anti* conformer (Fig. 2*A* lanes b-c). However, Mt-DdRP utilized A-coding of 8oA very inefficiently (Fig. 2*A* lanes b-c) and Cv-RdRP not at all (Fig. 2*A* lanes b-c). We initially anticipated that *syn*-8oA (as enol tautomer) would also pair with guanine and incorporate in place of cytidine (25). However, the incorporation of 8oA in place of cytidine was very inefficient and only observed for Ec-DdRP (Fig. 2*B* lanes b-c). In addition, a partial readthrough by two positions was observed upon waking Ec-DdRP through the AC sequence in the presence of 8oA, indicating an inefficient C-coding by 8oA (Fig. 2*A* lane b). Unexpectedly, all RNAPs in our set efficiently incorporated 8oA in place of uridine (Fig. 3*B* lanes b-c). We hypothesize that *syn*-8oA is responsible for the U-coding capacity (Fig. 3*B*). Noteworthy, such a conformer has to adopt an *imino* configuration to avoid clashes between the exocyclic amino groups of 8oA and adenine.

8oG was incorporated in place of guanine by all RNAPs in our set as expected from the coding capacity of the *anti* conformer (Fig. 3*A* lanes d-e). Ec-DdRP and Mt-DdRP also efficiently incorporated 8oG in place of uridine as expected from the coding capacity of the *syn* conformer (Fig. 3*B* lanes d-e) (22, 23). U-coding of 8oG was also evident from a partial readthrough by two positions during transcription of GU sequence (Fig. 3*A* lane d) and UG sequence (Fig. 3*B* lane d). Unexpectedly, Cv-RdRP failed to utilize U-coding of *syn*-8oG (Fig. 3*B* lanes d-e).

Interestingly, right-hand RNAPs efficiently utilized *anti*-8oG as a G-substitute (Fig. 3*A* lanes d-e) but failed to efficiently utilize *anti*-8oA as an A-substitute (Fig. 2*A* lanes d-e). While *anti*-8oG and *anti*-8oA retain all determinants to form canonical Watson–Crick base pairings with cytidine and uridine, respectively (Figs. 2*A* and 3*A*), oxo moieties disfavor *anti* conformations of nucleobases by clashing with 5′ phosphates and nucleo-sugars (22, 23, 25, 57). Presumably, right-hand RNAPs can cope with such distortions in case of *anti*-8oG that forms three hydrogen bonds with the acceptor cytidine (Fig. 3*A*), but not in case of *anti*-8oA that forms only two hydrogen bonds with the acceptor uridine or thymidine (Fig. 2*A*).

### Utilization of nucleoside analogs in processive transcription experiments

We next tested whether codings of nucleoside analogs identified in single-nucleotide addition experiments can be efficiently utilized in a broader sequence context by performing processive transcription experiments. In the case of DdRPs, we assembled EC as in the single-nucleotide addition experiments but using 70-nt-long DNA oligonucleotides. The resulting ECs contained 12 base pairs of upstream DNA, nine base pairs of RNA:DNA hybrid, and 49 base pairs of the DNA downstream of the primer-template junction. We used four different templates to test the utilization of A-, C-, G-, and U-codings of nucleoside analogs by DdRPs. The templates were designed not to contain the tested coding within the first several positions downstream of the primer-template junction. In the case of RdRP, we prepared a 71-nt-long RNA template by *in vitro* transcription with T7 RNAP and used a 24-nt-long RNA primer that formed 21 base pairs RNA:RNA duplex with the 3′ end of the RNA template. The resulting scaffold contained 50 nucleotides of the ssRNA template downstream of the primer-template junction. Given that RNA templates are more difficult to design and manufacture than DNA oligonucleotides, we used the same RNA template to test all four codings of nucleoside analogs utilized by Cv-RdRP.

**Figure 4:**
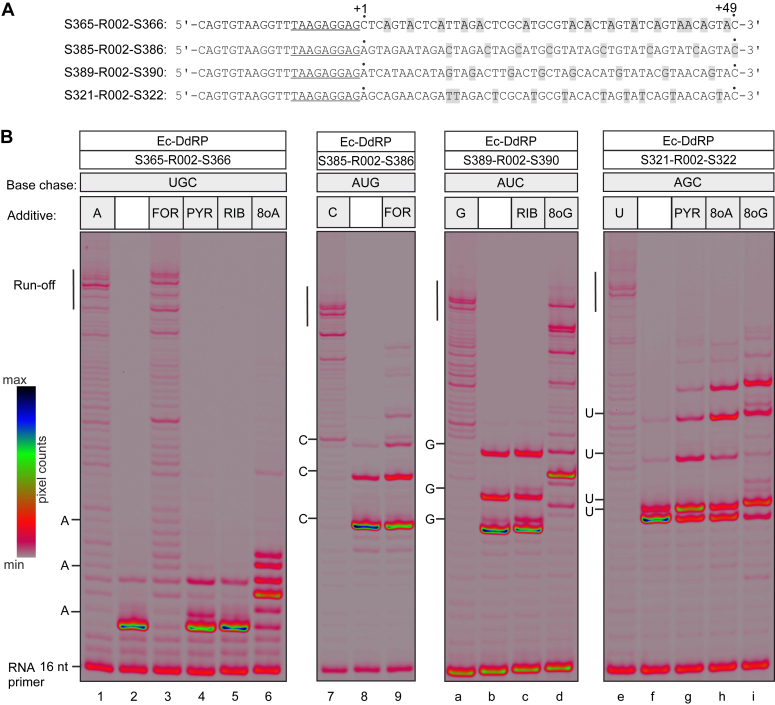
**Utilization of nucleoside analogs during processive transcription by Ec-DdRP**. *A*, schematics of nucleic acid scaffolds used to assemble ECs (only nontemplate DNAs are shown). The sequences corresponding to the annealing region of the RNA primer are *underlined*. Sequence positions encoding the nucleotide that is replaced by the nucleoside analog in utilization experiments are *highlighted*. *B*, assembled ECs were chased with four canonical NTPs (*lanes 1, 7a, and 7e*), or three canonical NTPs (*lanes 2, 8b, and 8f*), or three canonical NTPs and a triphosphorylated nucleoside analog (*lanes 3, 4, 5, 6, 9, c, d, g, h, and i*) for 5 min at 25 °C. NTPs and nucleoside analogs were added at 100 μM. 16 bit grayscale scans were normalized using max pixel counts within each gel panel and pseudocolored using RGB palette. The first three or four positions corresponding to the omitted canonical NTP are marked along the *left* edge of gel panels. Note that incorporation of 8oG results in a larger change of RNA mobility than incorporation of canonical NMPs, leading to misalignment of bands in neighboring lanes. All experiments were repeated in triplicate with similar results. 8oG, 8-oxoguanine; DdRP, DNA-dependent RNAP; EC, elongation complex.

**Figure 5:**
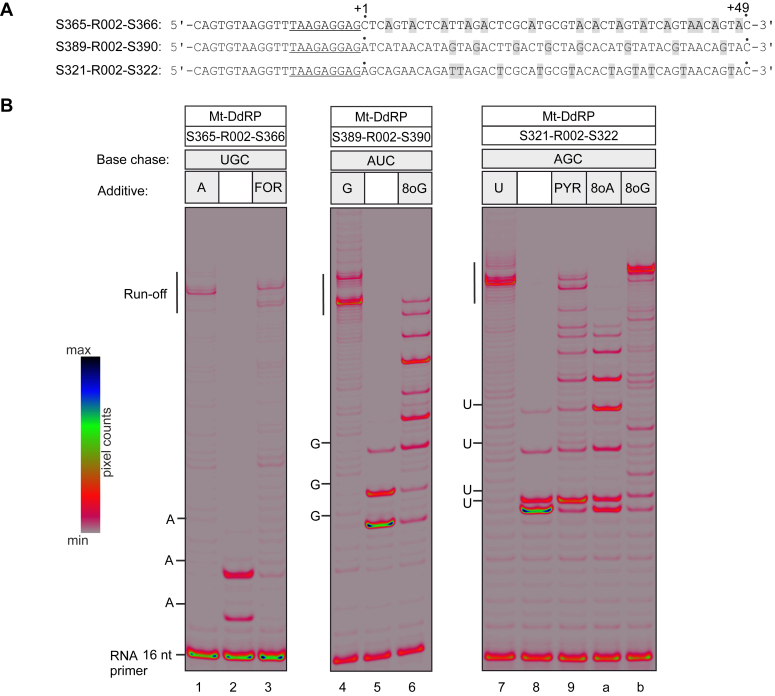
**Utilization of nucleoside analog during processive transcription by Mt-DdRP.***A*, schematics of nucleic acid scaffolds used to assemble ECs (only nontemplate DNAs are shown). The sequences corresponding to the annealing region of the RNA primer are *underlined*. Sequence positions encoding the nucleotide that is replaced by the nucleoside analog in utilization experiments are *highlighted*. *B*, assembled ECs were chased with four canonical NTPs (*lanes 1, 4, and 7*), or three canonical NTPs (*lanes 2, 5, and 8*), or three canonical NTPs and a triphosphorylated nucleoside analog (*lanes 3, 6, 9a and, 9b*) for 5 min at 25 °C. NTPs and nucleoside analogs were added at 100 μM. 16 bit grayscale scans were normalized using max pixel counts within each gel panel and pseudocolored using RGB palette. The first three or four positions corresponding to the omitted canonical NTP are marked along the *left* edge of gel panels. Note that incorporation of 8oG results in a larger change of RNA mobility than incorporation of canonical NMPs leading to misalignment of bands in neighboring lanes. All experiments were repeated in triplicate with similar results. 8oG, 8-oxoguanine; EC, elongation complex; Mt-DdRP, mitochondrial RNAP; NMP, nucleoside monophosphate.

**Figure 6:**
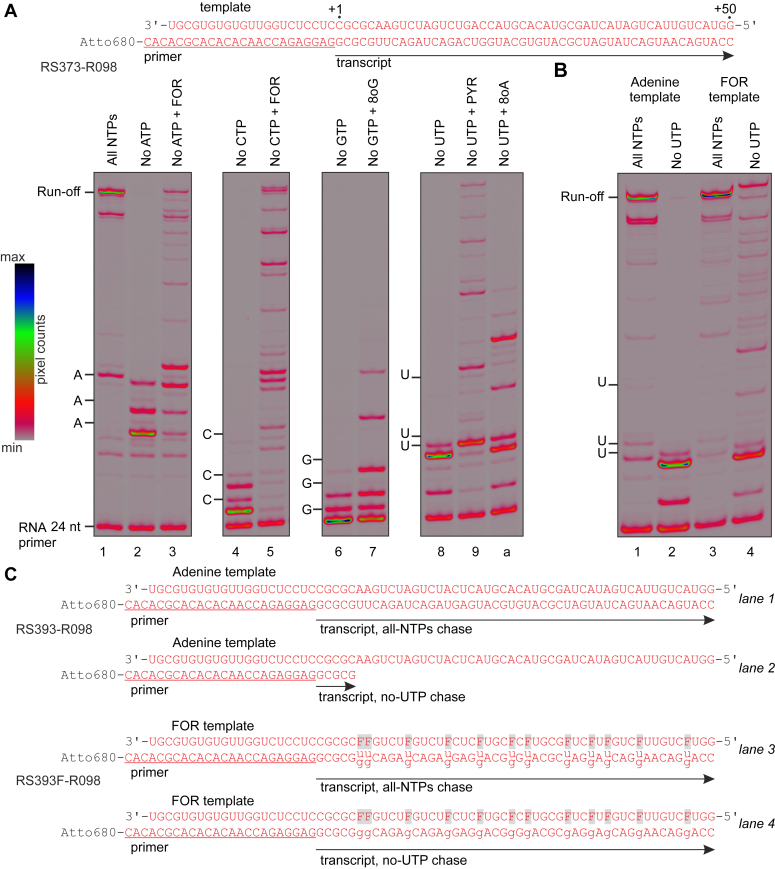
**Utilization of nucleoside analogs during processive transcription by Cv-RdRP.***A*, ECs were chased with four canonical NTPs (*lane 1*), or three canonical NTPs (*lanes 2, 4, 6, and8*), or three canonical NTPs and a triphosphorylated nucleoside analog (*lanes 3, 5, 7, and 9a*) for 5 min at 25 °C. Substrates were added at 100 μM. The first three positions corresponding to the omitted NTP are marked along the *left* edge of gel panels. Template RNA, RNA primer (*underlined*), and the RNA transcript are shown above the gel panels. *B*, experiments were performed as in (*A*) but using RNA templates synthesized using four canonical NTPs (*lanes 1, 2*) or CTP, GTP, UTP, and FOR (*lanes 3, 4*). *C*, schematics of experiments in (B). Positions of FOR in the template RNA are marked as “F”. Uridine or guanine may be incorporated against FOR in the all-NTPs chase, only guanine may be incorporated in the no-UTP chase. All experiments were repeated in triplicate with similar results. Cv-RdRP, RNAP from coxsackievirus; EC, elongation complex; FOR, formycin A.

When supplied with four canonical NTPs, all RNAPs in our set transcribed efficiently to the end of the employed templates synthesizing ∼50-nt-long segments of RNA (Figs. 4*B*, 5*B*, and 6*A*). When supplied with three canonical NTPs, RNAPs transcribed until the position coding for the missing NTP and paused or arrested strongly (Figs. 4*B*, 5*B*, and 6*A*). A fraction of RNAP was able to read through by misincorporation and paused strongly before the second position coding for the missing NTP. In some cases, a fraction of RNAP was able to reach the third position coding for the missing NTP. In all cases the readthrough beyond the third position coding for the missing NTP was extremely small. When supplied with three canonical NTPs and triphosphorylated nucleoside analogs, RNAPs transcribed further than with three canonical NTPs in several cases. In other cases, triphosphorylated nucleoside analogs did not improve transcription through positions coding for the missing NTP.

FOR in place of ATP allowed DdRPs to reach the end of the template almost as efficiently as in the presence of four canonical NTPs (Figs. 4*B* and 5*B* lane 3). Cv-RdRP was also able to reach the end of the template with FOR in place of ATP but paused or arrested strongly at several sites (Fig. 6*A* lane 3). When used in place of CTP, FOR only marginally improved transcription by Ec-DdRP; the bulk of RNAP was arrested within the first four positions coding for cytidine (Fig. 4*B* lane 9). In contrast, Cv-RdRP transcribed to the end of the template with FOR in place of CTP with approximately the same efficiency as when utilizing FOR in place of ATP (Fig. 6*A* lane 5).

PYR in place of ATP failed to improve transcription by Ec-DdRP (Fig. 4*B* lane 4). PYR was also a poor UTP substitute, the bulk of Ec-DdRP was arrested within the first five positions coding for uridine (Fig. 4*B* lane g). In contrast, right-hand RNAPs were able to reach the end of the template with PYR in place of UTP, albeit much less efficiently than when using four canonical NTPs (Figs. 5*B* and 6*A* lane 9). RIB failed to improve transcription by Ec-DdRP both as ATP and GTP substitute (Fig. 4*B* lanes 5, c).

8oG was a relatively efficient GTP substitute and a very poor UTP substitute for Ec-DdRP (Fig. 4*B* lanes d, i). In contrast, Mt-DdRP utilized 8oG in place of UTP very efficiently (Fig. 5*B* lane b) and transcribed to the end of the template with 8oG as GTP substitute, albeit rather inefficiently (Fig. 5*B* lane 6). 8oG in place of GTP allowed a fraction of Cv-RdRP to transcribe through three additional guanine coding positions compared to the GTP-less control experiment but was overall a poor GTP substitute for this enzyme (Fig. 6*A* lane 7).

8oA was a poor ATP substitute when utilized by Ec-DdRP; the transcription failed to proceed beyond three adenine coding positions (Fig. 4*B* lane 6). 8oA in place of UTP allowed Ec-DdRP and Cv-RdRP to transcribe through several additional uridine coding positions compared to the UTP-less control experiment but was overall a poor UTP substitute for these enzymes (Fig. 4*B* lane h; Fig. 6*A* lane a). While Mt-DdRP utilized 8oA as a UTP substitute more efficiently than Ec-DdRP and Cv-RdRP, it stopped short of reaching the end of the template within the 5-min timeframe of the experiment (Fig. 5*B* lane a).

Interestingly, while 8oA in place of ATP helped Ec-DdRP to transcribe through the first adenine coding position, it caused multiple arrests within five positions downstream (Fig. 4*B* lane 6). 8oA thus possibly interferes with the transcription elongation as a part of the nascent RNA:DNA hybrid within the EC. This effect may be related to the moderate inhibition of Cv-RdRP transcription by 8oA in a competitive mode, where 8oA is present at a 20-fold excess over the canonical NTPs (Fig. S5).

### FOR in the template RNA codes for uridine and guanine

The ability of FOR to dual code as a viral RdRP substrate is principally sufficient for a mutagenic effect on viral replication. However, we were interested in whether FOR can also dually code as an acceptor base in the template RNA, because such an ability is expected to double the mutagenic effect. FOR is incorporated very efficiently into RNA in place of adenine by T7-DdRP, which enabled us to use *in vitro* transcription to synthesize a template RNA-containing FOR in place of adenine. In the presence of four canonical NTPs, Cv-RdRP efficiently transcribed to the end of both the FOR-containing RNA template (Fig. 6*B* lane 3) and the control RNA template synthesized using canonical NTPs (Fig. 6*B* lane 1). When provided with ATP, CTP, and GTP but not UTP, Cv-RdRP stopped at the position coding for the first uridine when transcribing the control template (Fig. 6*B* lane 2). However, Cv-RdRP transcribed to the end of the FOR-containing template in the absence of UTP, albeit with a lower efficiency than when using all four canonical NTPs (Fig. 6*B* lane 4). These results indicate that Cv-RdRP can incorporate both uridine and guanine against the FOR acceptor base (Fig. 6*C*). If Cv-RdRP could not incorporate guanine against FOR, it would not be able to transcribe to the end of the FOR-containing template in the absence of UTP. If Cv-RdRP could not incorporate uridine in place of FOR, the omission of UTP should not have affected the overall efficiency of transcription of the FOR-containing template.

### Time-resolved measurements of FOR utilization by Cv-RdRP and Ec-DdRP

**Figure 7:**
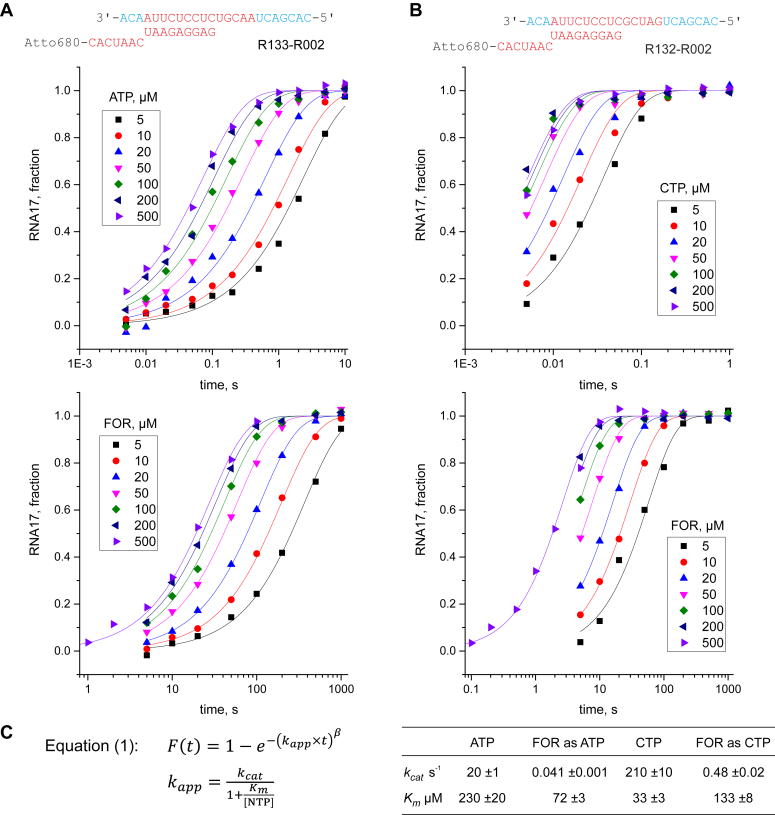
**Time-resolved measurements of FOR, ATP, and CTP utilization by Cv-RdRP.***A*, concentration series of adenine and FOR incorporation against uridine acceptor base. *B*, concentration series of cytidine and FOR incorporation against guanine acceptor base. *C*, FOR and CTP data were fit globally to a single exponential function, where the exponent follows the hyperbolic dependence on substrate concentration. ATP data were fit globally to a stretched exponential function (Equation 1), where the exponent follows the hyperbolic dependence on substrate concentration. The best-fit value of the stretching parameter β was 0.7. Errors are SDs of the best-fit estimates calculated by nonlinear regression of RNA17 fraction *versus* time using the Equation 1 and Origin 2015 software. Cv-RdRP, RNAP from coxsackievirus; DdRP, DNA-dependent RNAP; FOR, formycin A.

**Figure 8:**
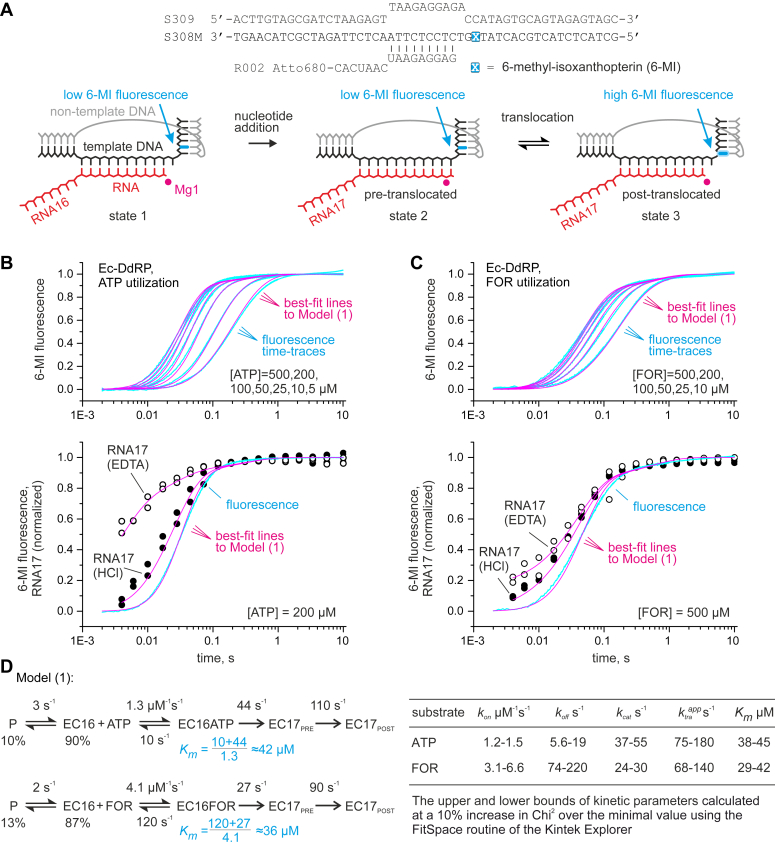
**Time-resolved measurements of ATP and FOR utilization by Ec-DdRP.***A*, schematics of the nucleic acid scaffold. The fluorescence of a guanine analog 6-MI (*cyan*) was quenched by neighboring base pairs in the initial EC (*state 1*) and the pretranslocated EC that formed following the nucleotide incorporation (*state 2*) but increased when the 6-MI relocated to the edge of the downstream DNA duplex upon translocation (*state 3*). *B*, ATP concentration series. *C*, FOR concentration series. *D*, kinetic model used for global analyses of FOR and AMP incorporation data. Compared to the schematics in (*A*), the model contained an additional species P (paused EC) to account for a small fraction (10–15%) of slowly reacting EC. The best-fit values of the reaction rates are indicated for each transition. 6-MI, 6-methyl-isoxanthopterin; DdRP, DNA-dependent RNAP; EC, elongation complex; FOR, formycin A.

Noting that Cv-RdRP incorporated FOR in place of adenine and cytidine with similar efficiency in processive transcription assays (Fig. 6*A* lanes 3, 5), we decided to investigate how FOR triphosphate compares with ATP and CTP in terms of the affinity for Cv-RdRP and the maximal incorporation rate (Fig. 7). We assembled ECs from chemically synthesized oligonucleotides and measured time courses of substrate incorporation using a rapid chemical quench-flow instrument for short time points (0.004–10 s) and manual mixing for longer durations (5–1000 s). Our data revealed that Cv-RdRP incorporated *syn*-FOR in place of CMP (0.5 s^−1^) and *anti*-FOR in place of AMP (0.04 s^-1^) 400 to 500 times slower than the corresponding canonical substrates (200 s^−1^ for CTP and 20 s^−1^ for ATP). The relatively slow rate of *syn*-FOR utilization was unsurprising considering that *syn*-FOR pair with *anti*-guanine has similar but not identical geometry to the canonical CG base pair. In contrast, the slow rate of *anti*-FOR utilization as A-substitute was an unexpected observation, considering that the geometries of adenine and *anti*-FOR base pairs with uridine acceptor base are nearly identical. Intrigued by these observations, we extended our investigation to Ec-DdRP. The kinetics of FOR incorporation by Ec-DdRP (Figs. 8 and S6) unveiled a contrasting picture, while FOR utilization as a C-substitute mirrored the slow rates observed with Cv-RdRP (0.8 s^−1^), the incorporation rate of FOR as an A-substitute (27 s^−1^) was on par with ATP utilization (44 s^−1^). This disparity suggests that the pyrazole moiety of FOR selectively impedes nucleotide incorporation by Cv-RdRP but not by Ec-DdRP, hinting at differences in the geometry of the reaction intermediates along the nucleotide incorporation pathway by these RNAPs.

## Discussion

In this study, we explored the utilization of nucleoside analogues featuring noncanonical nucleobases by three distinct RNAP families. Initially, we focused on the triphosphorylated forms of natural nucleoside analogues: FOR and PYR. Given the structural similarity between PYR and the synthetic nucleoside analog RIB, we broadened our analysis to include the latter. As our findings suggested that the incorporation of FOR and PYR into RNA likely involves the *syn* conformers of these nucleotides, we further extended our set of compounds to include oxidized purine nucleotides, 8oA and 8oG, which prior research indicated adopt *syn* conformations within the active sites of DNA polymerases.

### Substrate selection by three major families of RNAPs: a structural perspective

**Figure 9:**
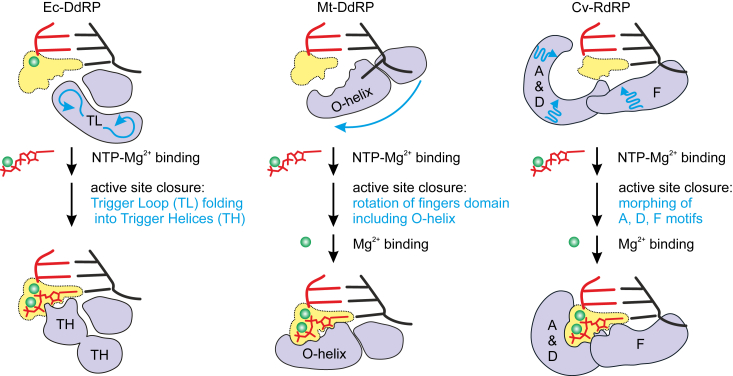
**The mechanism of NTP substrate loading and selection by diverse RNAPs.***A*, two-barrel DdRPs (*left*) feature largely preformed NTP-binding site (*yellow shapes*). Active site closure by mobile elements (*violet*) sterically inhibits NTP dissociation and activates catalysis by aligning the triphosphate moiety. Right-hand DdRPs (*middle*) and RdRPs (*right*) feature a composite NTP-binding site (*yellow shapes*) made of static and mobile (*violet*) structural elements. DdRP, DNA-dependent RNAP; RdRP, RNA-dependent representative; RNAP, RNA polymerase.

Ec-DdRP is a large multisubunit complex resembling a crab claw. The active site is formed at the interface of two double-psi-β-barrel structural elements, which lends the polymerase family its name (32, 33, 34, 35). Two-barrel DdRPs feature a largely preorganized active site with a tightly bound catalytic Mg^2+^ ion (Fig. 9). Upon recruitment, the substrate NTP in complex with a second Mg^2+^ ion assumes an inactive conformation. The closure of the active site by a mobile domain called the Trigger Loop (59, 60) activates the bound NTP by repositioning the triphosphate moiety for the inline attack by 3′OH of the RNA primer. Notably, base pairing interactions and nucleo-sugar recognition are established already upon the initial binding of the NTP substrate.

Both Mt-DdRP and Cv-RdRP are members of the right-hand family of nucleic acid polymerases. Their catalytic modules resemble the right hand: semiopen for DdRP, as if grasping a tennis ball (38, 61, 62), and semiclosed for RdRPs, akin to making an “OK” gesture (43, 44). Unlike the preorganized active site of two-barrel DdRPs, the active site of right-hand RNAPs assembles around the bound NTP and only recruits Mg^2+^ ions into their final catalytic positions during and after NTP binding (63, 64, 65, 66). Consequently, catalysis by right-hand RNAPs is likely more sensitive to proper positioning of nonbridging oxygens of the α-phosphate group of incoming NTP than in two-barrel enzymes (63). The extensive rearrangements of the active site residues upon NTP binding are referred to as the active site closure in both DNA- and RNA-dependent right-hand polymerases, but the details of the process are markedly different. In DdRPs, the active site closure involves a large-scale rotation of the fingers domain, which includes the structural element called O-helix (63, 66, 67) (Fig. 9, middle). In contrast, RdRPs undergo several smaller scale rearrangements, specifically in motifs A and D within the palm domain and motif F within the fingers domain (45, 46, 68) (Fig. 9, right).

RNAPs select the appropriate nucleobase with a preference ratio ranging from several hundred to several 1000-fold (52, 64, 69, 70). The selection of nucleobases by RNAPs involves at least two major determinants: (*i*) hydrogen bonding with the acceptor nucleobase, adhering to the Watson–Crick geometry, all while preserving the nucleo-sugar distances close to those found in canonical AU, AT, and CG base pairs and (*ii*) stacking interactions with the terminal nucleotide of the RNA primer. It is worth highlighting that potent stacking can facilitate the incorporation of substrates even in the absence of hydrogen bonding (71, 72). Moreover, it is plausible that the RNAP active site has evolved to stabilize specific unproductive pairings, like wobble base pairs, to diminish the chances of certain misincorporations, as has been shown for DNA polymerases (73).

Dissecting the contributions of individual amino acid residues to nucleobase selection proves challenging. This is due to the profound influence the active site closure has on selectivity across all RNAPs (64, 69, 71, 74, 75). Both two-barrel and right-hand RNAPs have evolved active sites that close stably only when the NTP correctly pairs with the template acceptor base. Alterations in the amino acid residues that influence the active site closure can yield enzymes with either enhanced catalytic activity at the expense of fidelity or *vice versa* (64, 76, 77, 78). Given these effects, it is no surprise that numerous residues are implicated in determining nucleobase selectivity.

### Differential utilization of nucleoside analogs by two-barrel and right-hand RNAPs

Our findings indicate that two-barrel RNAPs more readily utilize noncanonical base pairings in single-nucleotide addition experiments than right-hand RNAPs. Specifically, Ec-DdRP utilized up to 11 codings, while both Mt-DdRP and Cv-RdRP accepted only five codings each (Table 1). These observations are well in line with the fact that the active site is preorganized in two-barrel RNAPs but assembles around the bound NTP in right-hand enzymes (Fig. 9). Consequently, initial substrate binding in right-hand enzymes relies more heavily on nucleobase pairing with the acceptor nucleobase and its stacking interaction with the primer-terminal nucleotide.

**Table 1:** Utilization of nucleoside analogues in single-nucleotide addition experiments

RNAP	FOR in place of	PYR in place of	8oA in place of	8oG in place of	RIB in place of
Ec-DdRP	A, C, G	U, A	U, A	G, U	A, G
Mt-DdRP	A	U	U	G, U	
Cv-RdRP	A, C	U	U	G	

Contrastingly, right-hand RNAPs exhibited a superior capability to utilize noncanonical pairings in processive transcription experiments compared to Ec-DdRP. Ec-DdRP only managed to reach the end of the 49-nt transcript when utilizing codings mediated by canonical *anti*-*anti* pairings, specifically FOR as an A-substitute and 8oG as a G-substitute (Fig. 4*B* lanes 3,d). In comparison, Mt-DdRP utilized two codings mediated by noncanonical pairings of *syn* conformers: 8oG and PYR as U-substitutes (Fig. 5*B* lanes 9,b). Similarly, Cv-RdRP utilized FOR as a C-substitute and PYR as a U-substitute, both likely mediated by noncanonical pairings of *syn* conformers (Fig. 6*A* lanes 5, 9).

The limited ability of two-barrel RNAPs to utilize noncanonical codings during processive elongation can be attributed to at least two factors. First, two-barrel RNAPs possess a proofreading activity. Upon sensing a mismatch postincorporation, they can backtrack along the DNA (79, 80), leading to the extrusion of nascent RNA into the substrate loading channel (81, 82, 83, 84). This extruded RNA can be cleaved either by the intrinsic endonuclease activity of the RNAP active site (85, 86, 87) or more efficiently with auxiliary factors (88, 89, 90, 91) and specialized subunits (92). For instance, incorporating FOR instead of cytidine by Ec-DdRP triggers efficient cleavage of the RNA dinucleotide in a complex with extended RNA:DNA complementarity to allow backtracking (Fig. S7). Second, two-barrel RNAPs have evolved to respond to DNA-encoded signals such as regulatory pauses (93, 94). The introduction of noncanonical nucleotides into the nascent transcript might prompt Ec-DdRP to pause and, on occasion, backtrack, attempting to proofread the transcript. Unlike two-barrel RNAPs, most right-hand RNAPs lack both proofreading and intricate regulation by the transcribed sequence.

### Cv-RdRP uniquely rejects *syn*-8oG

Cv-RdRP was the only RNAP in our set that did not utilize *syn*-8oG as a U-substitute. A simple replacement of the substrate nucleobase with *syn*-8oG reveals that the exocyclic amino group is highly likely to clash with α-phosphate at some stage during the loading into the active site or in the transition state of the nucleotide addition reaction (Fig. S8*A*). We suggest this potential clash is the reason why Cv-RdRP fails to utilize 8oG as a U-substitute, whereas the clash may not occur or is tolerated during *syn*-8oG loading and incorporation by Mt-DdRP and Ec-DdRP.

A close inspection of our results also suggests that Cv-RdRP is in general highly sensitive to the crosstalk between the nucleobase and the α-phosphate of the substrate (Fig. S8*A*). For example, Cv-RdRP was less efficient than Mt-DdRP in utilizing *anti*-8oG as a G-substitute during processive transcription and the effect can be ascribed to clashes between the 8-oxo moiety of *anti*-8oG and its α-phosphate. Similarly, Cv-RdRP utilized *anti*-FOR ∼400 times slower than ATP (Fig. 7) and the effect can possibly be attributed to some form of interaction between the pyrazole moiety of *anti*-FOR and its α-phosphate. The latter interaction may involve hydrogen bonding or chelating of an additional Mg^2+^ ion.

In support of the latter hypothesis, the detailed quantitative dissection of ATP and FOR utilization kinetics by Ec-DdRP also revealed differences between the two substrates (Fig. 8). While Ec-DdRP incorporated AMP and FOR with similar turnovers and apparent affinities, our analysis suggests faster binding and dissociation rates for FOR (3.1–6.6 μM^−1^s^−1^ and 74–220 s^−1^) than ATP (1.2–1.5 μM^−1^s^−1^ and 5.6–19 s^−1^). These observations are consistent with the hypothesis that FOR is better preorganized for binding due to some form of interaction between the pyrazole moiety and the α-phosphate, yet the same interaction possibly interferes with subsequent accommodation and sequestration of FOR in the RNAP active site.

### Mt-DdRP uniquely rejects *syn*-FOR

Mt-DdRP was the only RNAP in our set that did not utilize *syn*-FOR as a C-substitute. We explored potential explanations for this selectivity. *Syn*-FOR shares the major groove edge (top edge in figures) with *syn*-8oA, a utilizable U-substitute for Mt-DdRP. In contrast, the minor groove edge (bottom edge in figures) of *syn*-FOR uniquely features pyrazole moiety that may interact with His1125 residue in Mt-DdRP (Fig. S8*B*). However, substitution of His1125 with alanine did not enable Mt-DdRP to incorporate *syn*-FOR against guanine (Fig. S8*C*), suggesting this residue is not crucial for discrimination. We then considered that Mt-DdRP could sense a minor clash between the exocyclic amino group of guanine and the pyrazole hydrogen of FOR (95). However, Mt-DdRP failed to incorporate *syn*-FOR against both guanine and inosine (the amino group-devoid counterpart of guanine), suggesting that the reason for rejection of *syn*-FOR lies elsewhere (Fig. S3). Our inability to pinpoint the exact reason for rejection of *syn*-FOR underscores the complexity of Mt-DdRP fidelity mechanism. For example, Mt-DdRP differs from Ec-DdRP and Cv-RdRP in that the acceptor nucleobase is not fully loaded into the active site when the active site is open (Fig. 9). It is uncertain how substrates are loaded in a sequence-specific manner. One possibility is that substrates are preloaded in the sequence independent manner and then are probed by the acceptor nucleobase that swings in and out of the active site (96). Another possibility is that the substrate is loaded in the “ajar” conformation where the acceptor nucleobase occupies an intermediate position between the catalytic and swing-out conformations (97, 98). Irrespective of the exact mechanism, we suggest that the intricate sequence of events accompanying substrate loading by Mt-DdRP in some way interferes with *syn*-FOR incorporation against guanine.

### Implications for the general principles of the nucleobase selectivity in RNAPs

Our findings illuminate the strategies that various RNAPs employ for selecting nucleobase analogs and canonical nucleobases. As illustrated in Figure 3*B*, the capability of a nucleobase to donate hydrogen to N1 of adenine within the Watson–Crick geometry primarily qualifies it to be identified as uridine. The lone exception was *syn*-FOR, which we perceive as indicative that the pyrazole hydrogen in *syn*-FOR predominantly localizes to a distinct nitrogen (Fig. S9). The hydrogen bond between N1H of guanine and a recipient atom in the substrate nucleobase is likely also pivotal in cytidine analog selection. We propose that *syn*-FOR is recognized as cytidine due to its dominant tautomer's ability to accept hydrogen from N1 of guanine (Fig. 2*B*). In contrast, *syn*-PYR is not identified as cytidine, because the pyrazole hydrogen localizes differently (Fig. S9).

Our study offers limited understanding of the selection of purine nucleotides. The purine analogs with incomplete rings, PYR and RIB, were not identified as purines by both Mt-DdRP and Cv-RdRP, emphasizing the vital role of stacking in purine recognition by right-hand RNAPs. In contrast, only Ec-RNAP, which has an intrinsically organized active site even before NTP binding (Fig. 9), incorporated PYR (Fig. 2*A* lanes 7–8) and RIB (Figs. 2*A* and 3*A* lanes 9-a) as substitutes for purines. Notably, Ec-RNAP did not recognize PYR as a G-substitute (Fig. 3*A*, lanes 7–8), suggesting that the carboxamide moiety of *anti*-PYR is preferentially oriented in an adenine-mimicking conformation (Fig. S10).

### Implications for the development of the nucleoside analog inhibitors of viral RdRPs

Viral RdRPs are targets for antiviral drugs, many of which are nucleoside analogs (99, 100). This is because viral RdRPs are often very compact so that their active site is the only druggable cavity. Two types of nucleoside analogs are investigated as antiviral drugs. Chain terminating nucleoside analogs with the modified nucleo-sugar utilize the differences in the nucleo-sugar selection mechanism between DdRPs (101, 102) and RdRP (45, 64, 103) to selectively incorporate and inhibit the latter (46, 104, 105). The second category is mutagenic nucleobases that rely on the differential effect of dual coding on DdRPs and RdRPs (20, 26, 27, 31). When incorporated by DdRPs, mutagenic nucleobases can cause translational errors and interfere with the functions of structural RNAs, whereas in the case of RdRPs they additionally cause inherited mutations in the viral genome. As a result, mutagenic nucleobases are more deleterious for the virus than for the host cell.

Nucleoside analogs hold the potential to serve as potent antivirals, particularly when they selectively disrupt RNA synthesis by viral RdRPs, while exhibiting minimal influence on transcription by nuclear and mitochondrial RNAPs. According to our findings, FOR is a competent dual coder for viral RdRP, supporting processive elongation through A- and C-codings in the NTP form and through U- and G-codings as an acceptor nucleobase in template RNA. This leads us to postulate that FOR may specifically target viral RdRP as a mutagenic base. However, we have observed that FOR is efficiently incorporated *in lieu* of adenine by both two-barrel and mitochondrial RNAPs. While it was not the focus of our current study, it is plausible that dual coding potential of FOR may interfere with the folding of structural RNAs and also disrupt the fidelity of translation (14, 106). Collectively, our findings suggest that the known toxicity of FOR (107, 108, 109) could be partially attributed to its postincorporation effects on the functioning of cellular RNAs.

A key revelation from our study is that C-coding of FOR is efficiently utilized by viral RdRP, less efficiently by two-barrel RNAPs and not at all by the mitochondrial RNAP. This suggests a possible strategy for enhancing the specificity of FOR by eliminating A-coding through nucleobase modifications at the nitrogen corresponding to N1 of adenine, thereby steering FOR predominantly toward viral RdRPs. If such a strategy is pursued, supplementary modifications to the nucleo-sugar would be necessary to render the compound inhibitory for viral RNA synthesis. For instance, the addition of a methyl group to the 2′C of the nucleo-sugar could convert the compound into a nonobligate chain terminator (46, 104, 105). An alternative approach might be to retain the mutagenic capacity of FOR's nucleobase but guide it toward viral RdRPs through nucleo-sugar modifications. Specifically, modifications like 1′-cyano have been shown to enhance the selectivity of nucleoside analogs for viral RdRPs (48, 110, 111). In summary, our findings suggest that FOR is a promising scaffold for the development of nucleoside analogs targeting viral RdRPs, potentially providing a novel route to effective antiviral treatments.

PYR is closely related to FOR in terms of biosynthetic logic but exhibits distinct transcriptional effects. Our experiments reveal that viral RdRP incorporates PYR solely as a U-substitute during processive elongation, albeit with modest efficiency. However, PYR also served as a moderately efficient U-substitute for the mitochondrial RNAP. While PYR demonstrated dual coding capabilities with the two-barrel RNAP, neither U- nor A-codings supported processive elongation. The limited incorporation and extension of PYR by all RNAPs tested likely arise from the suboptimal stacking interactions of its small, five-membered ring with adjacent nucleobases. While the stacking could potentially be improved by completing the second ring, such modifications could result in a FOR-like nucleobase. Our findings suggest that PYR may not serve as an ideal foundation for the development of nucleoside analogs targeting viral RdRP. This conclusion extends to RIB, which was incorporated by viral RdRP only after extended incubation with a high concentration of RIB triphosphate (Fig. S4). We also observed that PYR and RIB do not inhibit RNA synthesis by viral RdRP when present at 20-fold excess over the canonical NTPs and therefore do not function as competitive inhibitors (Fig. S5). In conclusion, we posit that a five-membered ring may be insufficient to create an effective RdRP substrate or a competitive inhibitor, even when adorned with substituents capable of mediating Watson–Crick base pairing. Nevertheless, it is plausible that compounds like PYR and RIB may serve as potent inhibitors of other enzymes involved in nucleic acid metabolism.

## Experimental procedures

### Reagents and oligonucleotides

DNA and RNA oligonucleotides were purchased from Eurofins Genomics GmbH and Fidelity Systems. DNA oligonucleotides and RNA primers are listed in Tables S1 and S2. NTPs were purchased from Thermo Fisher Scientific. 8oA, 8oG, and RIB triphosphates were purchased from Jena Bioscience. FOR and PYR nucleosides were purchased from Merck and triphosphorylated in-house (see below).

### Synthesis of for and PYR triphosphates

Freshly distilled phosphoryl chloride (18.8 μl, 0.202 mmol) and dry 2,4,6-trimethylpyridine (17.8 μl, 0.135 mmol) were added to a solution of FOR (36 mg, 0.135 mmol) in dry triethyl phosphate (0.8 ml) at −10 °C under nitrogen. The reaction was stirred at −10 °C for 3 h and at +4 °C overnight. A dry solution of tetrabutylammonium pyrophosphate (244 mg, 0.269 mmol) in MeCN (2 ml) and dry tributylamine (64 μl, 0.269 mmol) were added to the reaction mixture under nitrogen at 0 °C and the stirring was continued overnight at room temperature. The reaction was quenched by addition of 50 mM triethylammonium acetate buffer (4 ml) and chloroform (4 ml), and the stirring was continued for further 30 min. The aqueous phase was separated and washed with chloroform (3 ml). NaI (60 mg, 0.40 mmol) was added, and the solution was diluted with acetone (40 ml). The mixture was vortexed for 5 min and cooled at −20 °C for 30 min and the precipitated crude material was collected by centrifugation. The triphosphate product was purified by HPLC (Phenomemex Kinetex column, C18, 250 × 10 mm, 5 μm, flow rate 3 ml min^-1^) using a gradient of 50 mM triethylammonium acetate buffered water and acetonitrile (from 5% to 70% in 25 min). Lyophilization of the product containing fractions afforded the triethylammonium salt of the product. The FOR 5′-triphosphate was finally precipitated as the sodium salt (41 μmol, 30%) from cold, agitated solution of water (0.5 ml), NaI (40 mg) and acetone (13 ml). ^1^H NMR δH (500 MHz, D_2_O): 8.10 (s, H1, H-2), 5.29 (d, *J* = 6.9 Hz, 1H, H-1′), 4.66 (dd, *J* = 5.6 and 6.8 Hz, 1H, H-2′), 4.46 (dd, *J* = 4.2 and 5.6 Hz, 1H, H-3′), 4.32 (appq, *J* = 4.1 Hz, 1H, H4′), 4.28 – 4.18 (m, 2H, H5′ and H5′′). ^13^C NMR δC (125 MHz, D_2_O): 152.8 (C-6), 151.7 (C-2), 138.0 (C-9), 137.1 (C-4), 125.1 (C-5) 83.4 (d, *J*_C,*p*_ = 8.5 Hz, C-4′), 75.8 (C-1′), 74.1 (C-2′), 71.0 (C-3′), 65.6 (d, *J*_C,*p*_ = 5.5 Hz, C-5′). ^31^P NMR δP (202 MHz, D2O): −6.00 (d, *J* = 19.6 Hz, P-γ), −10.44 (d, *J* = 19.6 Hz, P-α), and – 21.27 (appt, *J* = 19.6 Hz, P-β). High resolution mass spectrometry (ESI^-^) *m/z*: [M-H]^-^ calcd for C_10_H_15_N_5_O_13_P_3_^-^ 505.9885, found 505.9890. The chemical structure validation data are presented in Figs. S11–S13. PYR (23 mg, 89 mmol) was converted to the 5′-triphosphate in an analogous manner as FOR. The product was purified by HPLC (Phenomemex Kinetex column, C18, 250 × 10 mm, 5 μm, flow rate 3 ml min-1) with 50 mM triethylammonium acetate buffered eluents and a gradient increasing from 3% MeCN to 70% in 25 min. The PYR 5′-triphosphate was finally precipitated as the sodium salt (8.1 μmol, 9%). PYR 5′-triphosphate is susceptible to spontaneous anomerization, which was evident during NMR measurements in D_2_O, affording a 10:1 mixture of *β*/*α* anomers overnight (see Fig. S13). The ratio of products was taken into consideration when calculating the concentration of the active anomer during polymerase assays. ^1^H NMR δH (500 MHz, D_2_O): 5.00 (d, *J* = 7.8 Hz, 1H, H-1′), 4.53 (dd, *J* = 5.4 and 7.7 Hz, 1H, H-2′), 4.45 (dd, *J* = 3.3 and 5.4 Hz, 1H, H-3′), 4.27 – 4.23 (m, 1H, H4′), and 4.23 – 4.15 (m, 2H, H5′ and H5′′). ^13^C NMR δC (125 MHz, D2O): 165.2 (C-6), 143.4 (C-4), 131.7 (C-5) 131.6 (C-3), 83.5 (d, *J*_C,*p*_ = 8.3 Hz, C-4′), 74.5 (C-1′), 73.7 (C-2′), 71.1 (C-3′), and 65.6 (d, *J*_C,*p*_ = 5.5 Hz, C-5′). ^31^P NMR δP (202 MHz, D_2_O): −6.45 (d, *J* = 19.6 Hz, P-γ), −10.71 (d, *J* = 20.0 Hz, P-α), and – 21.95 (appt, *J* = 20.0 Hz, P-β). High resolution mass spectrometry (ESI^-^) *m/z*: [M-H]^-^ calcd for C_9_H_15_N_3_O_15_P_3_^-^ 497.9721, found 497.9723. The chemical structure validation data are presented in Figs. S11–S13.

The molar amounts of nucleoside triphosphates were measured by quantitative NMR. ^1^H-NMR spectra were acquired with relaxation delay (d1) of 30 s and using acetonitrile as an internal standard.

### Protein expression and purification

Plasmids used for protein expression are listed in Table S3. Ec-DdRP was expressed in *E. coli* T7 Express *lysY/I*^*q*^ (New England Biolabs) bearing the pVS10 plasmid and purified by Ni-, heparin, and Q-sepharose chromatography as described previously (112). Mt-DdRP lacking the mitochondrial localization signal was expressed in *E. coli* T7 Express *lysY/I*^*q*^ bearing the pRP009 plasmid and purified by Ni-, Hepari,n and S-sepharose chromatography as described previously (21, 49). *E. coli* GreA was expressed in *E. coli* T7 Express *lysY/I*^*q*^ cells bearing the pIA578 plasmid and purified by Ni-sepharose, followed by gel filtration as described previously (113). The protein was dialyzed against 50% glycerol, 20 mM Tris–HCl pH 7.9, 1 M NaCl, 0.1 mM EDTA, 0.1 mM DTT and stored at −20 °C.

Cv-RdRP was expressed in *E. coli* T7 Express *lysY/I*^*q*^ bearing the pGB161 plasmid encoding a fusion of six-histidine tag, GB1 solubility domain, Tobacco Etch Virus (TEV) protease site followed by Cv-RdRP. The cells were grown in LB medium supplemented with 30 μg/ml kanamycin at 30 °C until *A*_600_ ∼1, the culture was transferred to 22 °C, and protein expression was induced for 10 h by the addition of 1 mM IPTG. Cells were harvested by centrifugation at 7000*g* for 10 min at 4 °C and stored at −80 °C. Cell pellet was resuspended in the lysis buffer (50 mM Tris–HCl pH 7.9, 500 mM NaCl, 5% glycerol, and 1 mM β-mercaptoethanol) supplemented with one tablet of EDTA-free protease inhibitors (Roche Applied Science) per 50 ml of buffer and 1 mg/ml lysozyme. Cells were incubated on ice for 45 min and disrupted by sonication. The lysate was cleared by centrifugation at 58,000*g* for 50 min at 5 °C. The supernatant was supplemented with 10 mM imidazole and loaded onto a 1 ml Ni-sepharose (GE Healthcare) gravity column pre-equilibrated with lysis buffer. Protein was eluted using a step gradient (30, 60, 300 mM) of imidazole in lysis buffer. The 300 mM imidazole fraction containing Cv-RdRP was treated with TEV protease for 8 h at 6 °C to remove six-histidine tag and GB1 solubility tag. TEV treated protein was diluted 3-fold with buffer A (50 mM Tris–HCl pH 7.9, 5% glycerol, 1 mM β-mercaptoethanol, and 0.1 mM EDTA) and loaded onto 6 ml Resource Q column (GE Healthcare) equilibrated with buffer A and eluted with a gradient of buffer B (buffer A supplemented with 1.5 M NaCl). Cv-RdRP eluted from the Resource Q column at > 20% buffer B. Cv-RdRP was purified further by gel filtration using 120 ml HiPrep 16/60 Sephacryl S-200 HR column in buffer A supplemented with 0.5 M NaCl. Cv-RdRP eluted from the gel filtration column at ∼55 to 60 ml. The fractions containing the purified protein were pooled together, concentrated using Amicon Ultra-4 centrifugal filter (Merck Milipore), diluted by adding two volumes of 1.5× storage buffer (75% glycerol, 30 mM Tris–HCl pH 7.9, 225 mM NaCl, 0.15 mM EDTA, and 0.75 mM DTT) to one volume of Cv-RdRP preparation and stored at −80 °C.

T7 RNAP with performance enhancing P266L substitution was expressed in *E. coli* T7 Express *lysY/I*^*q*^ bearing the pBH161-P266L plasmid (114). The cells were grown in LB medium supplemented with 100 μg/ml carbenicillin at 30 °C until *A*_600_ ∼1, the culture was transferred to 22 °C, and protein expression was induced for 10 h with 1 mM IPTG. Cells were harvested by centrifugation at 7000*g* for 10 min at 4 °C and stored at −80 °C. Cell pellet was resuspended in the lysis buffer supplemented with one tablet of EDTA-free protease inhibitors (Roche Applied Science) per 50 ml of buffer and 1 mg/ml lysozyme. Cells were incubated on ice for 45 min and disrupted by sonication. The lysate was cleared by centrifugation at 58,000*g* for 50 min at 5 °C. The supernatant was supplemented with 10 mM imidazole and loaded onto a Ni-sepharose column pre-equilibrated with the lysis buffer. Protein was eluted using a step gradient (30, 60, 300 mM) of imidazole in the lysis buffer. The 300 mM imidazole fraction containing T7 RNAP was diluted 3-fold with buffer A, loaded onto a 5 ml Heparin column (GE Healthcare) equilibrated with buffer A, and eluted with a gradient of buffer B. T7 RNAP eluted at > 25% buffer B. Fractions containing T7 RNAP were pooled, diluted 3-fold with buffer A, loaded onto a 6 ml Resource Q column equilibrated with buffer A, and eluted with a gradient of buffer B. T7 RNAP eluted from the Resource Q column at > 12% buffer B. The fractions containing the purified protein were pooled together, concentrated using Amicon Ultra-4 centrifugal filters (Merck Milipore), and diluted by adding two volumes of 1.5× storage buffer (75% glycerol, 30 mM Tris–HCl pH 7.9, 225 mM NaCl, 0.15 mM EDTA, and 0.75 mM DTT) to one volume of the T7 RNAP preparation and stored at −80 °C.

### Synthesis and purification of long RNA templates for Cv-RdRP

RNA templates (RS373 and RS393, 70-nt long, Table S2) were synthesized by *in vitro* transcription of chemically synthesized dsDNA templates (oligonucleotides S373-S374 and S393-S394, Table S1) by in-house purified T7 RNAP containing P266L amino acid substitution. The reaction mixes (0.1–1 ml) contained 0.2 μM DNA template, 1.0 μM T7 RNAP, 0.2 μM yeast inorganic pyrophosphatase, 2.3 mM ATP, CTP, GTP, UTP (or FOR triphosphate in place of ATP where indicated), 40 mM Hepes–KOH pH 7.5, 10 mM MgCl_2_, 80 mM KCl, 5% glycerol, 5 mM DTT, and 0.1 mM EDTA. Reactions were incubated for 3 h at 37 °C, supplemented with 0.02 units/μl of RNase free DNase I (Thermo Fisher Scientific) and incubated for an additional 20 min at 25 °C. Reactions were loaded onto Capto HiRes Q 5/50 anion exchange column (Cytiva) equilibrated with buffer NA (50 mM Tris–HCl pH 7.9, 5% glycerol, 1 mM β-mercaptoethanol, 1 mM EDTA) and eluted with a gradient of buffer NB (Buffer NA supplemented with 1.5 M NaCl). RNA eluted at > 35% of buffer NB. Fractions with the elevated UV absorbance were evaluated on 1% agarose gel in 1xTBE buffer. Fractions containing RNA were pooled and mixed with 0.6-fold volume of 100% isopropanol and incubated for 20 min at 22 °C. RNA was precipitated by centrifugation at 21,000*g* for 10 min at 4 °C. RNA pellets were supplemented with 1 ml of 70% (v:v) ethanol and centrifuged at 15,000*g* for 5 min at 22 °C. Supernatant was discarded; pellets were dried and dissolved in 10 mM Hepes–KOH pH 7.5, 0.1 mM EDTA. The typical yield was 100 μl of ∼50 μM RNA template per 100 μl of the *in vitro* transcription mix containing four canonical NTPs. The typical yield was 50 μl of ∼25 μM RNA template per 100 μl of the *in vitro* transcription mix containing CTP, GTP, UTP, and FOR triphosphate.

### *In vitro* transcription reactions and single-nucleotide addition assays

Four-part ECs containing template DNA, RNA primer, nontemplate DNA (oligonucleotides are listed in Tables S1 and S2), and DdRPs were assembled by a procedure developed by Komissarova *et al.* 2003 (115) in TB10 buffer (40 mM Hepes–KOH pH 7.5, 10 mM MgCl_2_, 80 mM KCl, 5% glycerol, 0.1 mM EDTA, and 0.1 mM DTT). RNA primer (1 μM) was annealed to the template DNA (1.4 μM), incubated with DdRP (1.5 μM) for 10 min, and then with the nontemplate DNA (2 μM) for 20 min at 25 °C. Three-part ECs containing template RNA, RNA primer, and Cv-RdRP were assembled in VTB0 buffer (40 mM Hepes–KOH pH 7.5, 5% glycerol, 0.1 mM EDTA, and 5 mM DTT). RNA primer (1 μM) was annealed with template RNA (1.4 μM) and incubated with Cv-RdRP (2.0 μM) for 20 min at 25 °C.

The transcription reactions were initiated by the addition of 10 μl of NTPs solution to 10 μl of the assembled EC solution. Both solutions were prepared in a transcription buffer. TB10 buffer was used for DdRPs and VTB10 buffer (40 mM HEPES–KOH pH 7.5, 10 mM MgCl_2_, 5% glycerol, 0.1 mM EDTA, and 5 mM DTT) was used for Cv-RdRP. The final reaction mixes contained 0.1 μM EC, 10 μM cognate NTPs (where present), 100 μM noncognate NTPs (where present), and 100 μM NTP analogs (where present). The reactions were incubated for 1 min at 25 °C and quenched with 30 μl of gel loading buffer (94% formamide, 20 mM Li_4_-EDTA, and 0.2% Orange G). RNAs were separated on 16% denaturing polyacrylamide gels and visualized with an Odyssey Infrared Imager (Li-Cor Biosciences); band intensities were quantified using the ImageJ software (https://imagej.net/ij/) (116).

Noteworthy, at the employed reactant concentrations (0.1 μM EC, 100 μM NTP substrate), a 0.1% contamination of a nonincorporating substrate with the cognate substrate is stoichiometrically sufficient for the complete extension of the EC with the contaminating substrate. Fig. S14 summarizes our efforts to rule out the possibility that RNAPs utilized contaminating cognate NTPs instead of nucleoside analogs to extend ECs.

### *In vitro* transcription reactions and processive transcription

Four-part ECs containing template DNA, RNA primer, nontemplate DNA (oligonucleotides are listed in Tables S1 and S2) and DdRPs were assembled in TB0 buffer (40 mM Hepes–KOH pH 7.5, 80 mM KCl, 5% glycerol, 0.1 mM EDTA, and 0.1 mM DTT). RNA primer (1 μM) was annealed to the template DNA (1.4 μM), incubated with DdRP (1.5 μM) for 10 min, and then with the nontemplate DNA (2 μM) for 20 min at 25 °C. Three-part ECs containing template RNA, RNA primer, and Cv-RdRP were assembled in VTB0 buffer. RNA primer (1 μM) was annealed with template RNA (1.4 μM) and incubated with Cv-RdRP (2.0 μM) for 20 min at 25 °C.

The transcription reactions were initiated by the addition of 5 μl of assembled EC to 5 μl of NTPs mixture. In case of DdRPs, EC solutions were prepared in TB0 buffer and NTP mixes were prepared in TB20 buffer (40 mM Hepes–KOH pH 7.5, 20 mM MgCl_2_, 80 mM KCl, 5% glycerol, 0.1 mM EDTA, and 0.1 mM DTT). In case of Cv-RdRP, EC solutions were prepared in VTB0 buffer and NTP mixes were prepared in VTB20 buffer (40 mM Hepes–KOH pH 7.5, 20 mM MgCl_2_, 5% glycerol, 0.1 mM EDTA, and 5 mM DTT). The final reaction mixes contained 0.1 μM ECs. The concentrations of NTPs and NTP analogs were 0 μM for a zero-control reaction and 100 μM each for 3-NTPs or 4-NTPs reactions. In a competitive setup, the concentration of NTP analogs was 2 mM. The reactions were incubated for 5 min at 25 °C and quenched with 20 μl of gel loading buffer. RNAs were separated on 16% denaturing polyacrylamide gels, visualized, and quantified as described above.

In the case of DdRPs, the run-offs consisted of multiple bands. One likely reason is the heterogeneity of 5′ ends of the HPLC purified oligonucleotides utilized as template DNAs. Appearance of RNA products longer than DNA templates was likely due to DdRPs switching to a new template when reaching the end of DNA (117, 118, 119). Nevertheless, the multiband nature of run-offs did not affect the interpretations of the processive transcription data.

### GreA facilitated RNA cleavage

Four-part ECs containing template DNA, RNA primer, nontemplate DNA (oligonucleotides are listed in Tables S1 and S2) and Ec-DdRP were assembled in TB1 buffer (40 mM Hepes–KOH pH 7.5, 1 mM MgCl_2_, 80 mM KCl, 5% glycerol, 0.1 mM EDTA, and 0.1 mM DTT). RNA primer (1 μM) was annealed to the template DNA (1.4 μM), incubated with Ec-DdRP (1.5 μM) for 10 min, and then with the nontemplate DNA (2 μM) for 20 min at 25 °C. Extended ECs were prepared by incubating the assembled EC (1 μM) with 50 μM UTP and GTP or 100 μM PYR triphosphate and 50 μM GTP in TB1 buffer for 3 min at 25 °C and passed through Zeba Spin desalting columns 40K MWCO (Pierce Biotechnology) pre-equilibrated with TB0 buffer. RNA cleavage was initiated by mixing 80 μl of the 0.2 μM EC in TB0 buffer with 80 μl of 20 μM GreA in TB20 buffer (40 mM Hepes–KOH pH 7.5, 20 mM MgCl_2_, 80 mM KCl, 5% glycerol, 0.1 mM EDTA, and 0.1 mM DTT) at 25 °C. The final reaction mixture contained 0.1 μM EC, 10 μM GreA, and 10 mM Mg^2+^. Twenty microliters aliquots were withdrawn at the indicated time points and quenched with 40 μl of gel loading buffer. RNAs were separated on 16% denaturing polyacrylamide gels, visualized, and quantified as described above.

### Time-resolved nucleotide addition measurements

Time-resolved measurements of nucleotide addition were performed in an RQF-3 quench-flow instrument (KinTek Corporation) or by manual mixing for longer durations. When RQF-3 was used, the reaction was initiated by the rapid mixing of 14 μl of 0.2 μM EC with 14 μl of 2× substrate solution. Ec-DdRP ECs were prepared in TB0 and 2× substrate solutions were prepared in TB20 buffer. Cv-DdRP ECs were prepared in VTB0 buffer and 2× substrate solutions were prepared in VTB20 buffer. The reaction was allowed to proceed for 0.004 to 10 s at 25 °C and quenched by the addition of 86 μl of 0.45 M EDTA or 0.5 M HCl. EDTA quenched reactions were mixed with 171 μl of gel loading buffer. HCl quenched reactions were immediately neutralized by adding 171 μl of Neutralizing-Loading Buffer (94% formamide, 290 mM Tris base, 13 mM Li_4_-EDTA, 0.2% Orange G). When manual mixing was used, 75 μl of 0.2 μM EC was mixed with 75 μl of 2× substrate solutions, then 14 μl aliquots were withdrawn at the indicated time points and quenched with 43 μl for 0.5 M HCl. Reactions were immediately neutralized by adding 86 μl of neutralizing-loading buffer. RNAs were separated on 16% denaturing polyacrylamide gels, visualized, and quantified as described above.

### Time-resolved fluorescence measurements

Measurements were performed in SFM-3000 stopped-ﬂow instruments (Biologic, Seyssinet-Pariset) equipped with μFC-08 cuvette at 25 °C. Ec-DdRP ECs were assembled on nucleic acids scaffold composed of chemically synthesized oligonucleotides. The fluorescent guanine analog 6-methyl-isoxanthopterin (6-MI) was initially positioned in the downstream DNA two nucleotides downstream of the active site (21). The 6-MI fluorescence was quenched by the neighboring base pairs in the initial EC and the pretranslocated EC that formed following the nucleotide incorporation but increases when the 6-MI relocates to the edge of the downstream DNA upon translocation. The 6-MI fluorophore was excited at 340 nm, and the emitted light was collected through a 400 nm longpass filter. The nucleotide addition reactions were initiated by mixing 75 μl of 0.2 μM EC with 75 μl of 2× substrate solution. Both solutions were prepared in TB10 buffer. At least three individual traces were averaged for each concentration of the substrate.

### Data analyses

Time-resolved nucleotide incorporation concentration series of Cv-RdRP (RNA17 time points) were globally fitted to a single exponential (CTP and FOR in place of ATP datasets) or stretched exponential (ATP and FOR in place of CTP datasets) function using Origin 2015 software (https://www.originlab.com/) (OriginLab): the exponent followed a hyperbolic dependence on the substrate concentration; Michaelis constant *Km*, rate constant *k*_*cat*_*;* and the stretching parameter β were shared by all curves in the dataset.

Time-resolved AMP and CMP incorporation data by Ec-DdRP (HCl and EDTA quenched reactions) and the translocation timetraces were simultaneously fitted to a four-step model using the numerical integration capabilities of the Kin-Tek Explorer software (https://www.kintekexplorer.com/) (KinTek Corporation) (120). The model postulated that the initial EC16 equilibrates with a small fraction of reversibly inactivated EC (designated P for paused EC). Upon mixing with NTP solution, EC16 reversibly binds the substrate, undergoes the irreversible transition to EC17 upon incorporation of the nucleotide into RNA, followed by the irreversible translocation. The EDTA quenched reactions were modeled using the pulse-chase routine of the Kin-Tek Explorer software. A detailed description of the data analysis routines is presented in (102).

## Data availability

All data are included in the article, supporting information, and the supplementary data file.

## Supporting information

This article contains supporting information (49, 113, 114, 121, 122).

## Declaration of generative AI and AI-assisted technologies in the writing process

During the preparation of this work, the authors used ChatGPT and Gemini to improve the language and readability. After using these tools, the authors reviewed and edited the content as needed and take full responsibility for the content of the publication.

## Conﬂict of interests

The authors declare that they have no conflicts of interest with the contents of this article.

## References

[1] Wang S.-A., Ko Y., Zeng J., Geng Y., Ren D., Ogasawara Y. (2019). Identification of the Formycin A Biosynthetic Gene Cluster from Streptomyces kaniharaensis Illustrates the Interplay between Biological Pyrazolopyrimidine Formation and de Novo Purine Biosynthesis. J. Am. Chem. Soc..

[2] Zhang M., Zhang P., Xu G., Zhou W., Gao Y., Gong R. (2020). Comparative investigation into formycin A and pyrazofurin A biosynthesis reveals branch pathways for the construction of C-nucleoside scaffolds. Appl. Environ. Microbiol..

[3] De Clercq E. (2016). C-nucleosides to Be revisited. J. Med. Chem..

[4] Hultin P.G. (2005). Bioactive C-glycosides from bacterial secondary metabolism. Curr. Top. Med. Chem..

[5] Isono K. (1988). Nucleoside antibiotics: structure, biological activity, and biosynthesis. J. Antibiot..

[6] Graci J.D., Cameron C.E. (2006). Mechanisms of action of ribavirin against distinct viruses. Rev. Med. Virol..

[7] Ward D.C., Reich E. (1968). Conformational properties of polyformycin: a polyribonucleotide with individual residues in the syn conformation. Proc. Natl. Acad. Sci. U. S. A..

[8] Prusiner P., Brennan T., Sundaralingam M. (1973). Crystal structure and molecular conformation of formycin monohydrates. Possible origin of the anomalous circular dichroic spectra in formycin mono- and polynucleotides. Biochemistry.

[9] DeWolf W.E., Fullin F.A., Schramm V.L. (1979). The catalytic site of AMP nucleosidase. Substrate specificity and pH effects with AMP and formycin 5’-PO4. J. Biol. Chem..

[10] Dodin G., Bensaude O., Dubois J.E. (1980). Tautomerism of formycin. Mechanism of interconversion. J. Am. Chem. Soc..

[11] Włodarczyk J., Stoychev Galitonov G., Kierdaszuk B. (2004). Identification of the tautomeric form of formycin A in its complex with Escherichia coli purine nucleoside phosphorylase based on the effect of enzyme-ligand binding on fluorescence and phosphorescence. Eur. Biophys. J..

[12] Rossomando E.F., Jahngen J.H., Eccleston J.F. (1981). Formycin 5’-triphosphate, a fluorescent analog of ATP, as a substrate for adenylate cyclase. Proc. Natl. Acad. Sci. U. S. A..

[13] Koellner G., Bzowska A., Wielgus-Kutrowska B., Luić M., Steiner T., Saenger W. (2002). Open and closed conformation of the E. coli purine nucleoside phosphorylase active center and implications for the catalytic mechanism. J. Mol. Biol..

[14] Ward D.C., Cerami A., Reich E., Acs G., Altwerger L. (1969). Biochemical studies of the nucleoside analogue, formycin. J. Biol. Chem..

[15] Gutowski G.E., Sweeney M.J., DeLong D.C., Hamill R.L., Gerzon K., Dyke R.W. (1975). Biochemistry and biological effects of the pyrazofurins (pyrazomycins): initial clinical trial. Ann. N. Y. Acad. Sci..

[16] Cadman E., Benz C. (1980). Uridine and cytidine metabolism following inhibition of de novo pyrimidine synthesis by pyrazofurin. Biochim. Biophys. Acta.

[17] Dix D.E., Lehman C.P., Jakubowski A., Moyer J.D., Handschumacher R.E. (1979). Pyrazofurin metabolism, enzyme inhibition, and resistance in L5178Y cells. Cancer Res..

[18] Dye F.J., Rossomando E.F. (1982). *In vitro* processing of the adenosine analog formycin A to the mono-, di-, and triphosphate by a soluble multienzyme system from mouse liver. Biosci. Rep..

[19] Darlix J.L., Fromageot P., Reich E. (1971). Analysis of transcription in vitro using purine nucleotide analogs. Biochemistry.

[20] Crotty S., Maag D., Arnold J.J., Zhong W., Lau J.Y., Hong Z. (2000). The broad-spectrum antiviral ribonucleoside ribavirin is an RNA virus mutagen. Nat. Med..

[21] Prajapati R.K., Rosenqvist P., Palmu K., Mäkinen J.J., Malinen A.M., Virta P. (2019). Oxazinomycin arrests RNA polymerase at the polythymidine sequences. Nucleic Acids Res..

[22] Hsu G.W., Ober M., Carell T., Beese L.S. (2004). Error-prone replication of oxidatively damaged DNA by a high-fidelity DNA polymerase. Nature.

[23] Brieba L.G., Eichman B.F., Kokoska R.J., Doublié S., Kunkel T.A., Ellenberger T. (2004). Structural basis for the dual coding potential of 8-oxoguanosine by a high-fidelity DNA polymerase. EMBO J..

[24] Kamiya H., Miura H., Murata-Kamiya N., Ishikawa H., Sakaguchi T., Inoue H. (1995). 8-Hydroxyadenine (7,8-dihydro-8-oxoadenine) induces misincorporation in in vitro DNA synthesis and mutations in NIH 3T3 cells. Nucleic Acids Res..

[25] Koag M.-C., Jung H., Lee S. (2019). Mutagenic replication of the major oxidative adenine lesion 7,8-Dihydro-8-oxoadenine by human DNA polymerases. J. Am. Chem. Soc..

[26] Gordon C.J., Tchesnokov E.P., Schinazi R.F., Götte M. (2021). Molnupiravir promotes SARS-CoV-2 mutagenesis *via* the RNA template. J. Biol. Chem..

[27] Kabinger F., Stiller C., Schmitzová J., Dienemann C., Kokic G., Hillen H.S. (2021). Mechanism of molnupiravir-induced SARS-CoV-2 mutagenesis. Nat. Struct. Mol. Biol..

[28] Wood M.L., Esteve A., Morningstar M.L., Kuziemko G.M., Essigmann J.M. (1992). Genetic effects of oxidative DNA damage: comparative mutagenesis of 7,8-dihydro-8-oxoguanine and 7,8-dihydro-8-oxoadenine in Escherichia coli. Nucleic Acids Res..

[29] Tan X., Grollman A.P., Shibutani S. (1999). Comparison of the mutagenic properties of 8-oxo-7,8-dihydro-2’-deoxyadenosine and 8-oxo-7,8-dihydro-2’-deoxyguanosine DNA lesions in mammalian cells. Carcinogenesis.

[30] Kamiya H. (2003). Mutagenic potentials of damaged nucleic acids produced by reactive oxygen/nitrogen species: approaches using synthetic oligonucleotides and nucleotides: survey and summary. Nucleic Acids Res..

[31] Pauly M.D., Lauring A.S. (2015). Effective lethal mutagenesis of influenza virus by three nucleoside analogs. J. Virol..

[32] Iyer L.M., Koonin E.V., Aravind L. (2003). Evolutionary connection between the catalytic subunits of DNA-dependent RNA polymerases and eukaryotic RNA-dependent RNA polymerases and the origin of RNA polymerases. BMC Struct. Biol..

[33] Cramer P., Bushnell D.A., Kornberg R.D. (2001). Structural basis of transcription: RNA polymerase II at 2.8 angstrom resolution. Science.

[34] Zhang G., Campbell E.A., Minakhin L., Richter C., Severinov K., Darst S.A. (1999). Crystal structure of Thermus aquaticus core RNA polymerase at 3.3 A resolution. Cell.

[35] Vassylyev D.G., Sekine S., Laptenko O., Lee J., Vassylyeva M.N., Borukhov S. (2002). Crystal structure of a bacterial RNA polymerase holoenzyme at 2.6 A resolution. Nature.

[36] Werner F., Grohmann D. (2011). Evolution of multisubunit RNA polymerases in the three domains of life. Nat. Rev. Microbiol..

[37] Cermakian N., Ikeda T.M., Miramontes P., Lang B.F., Gray M.W., Cedergren R. (1997). On the evolution of the single-subunit RNA polymerases. J. Mol. Evol..

[38] Ringel R., Sologub M., Morozov Y.I., Litonin D., Cramer P., Temiakov D. (2011). Structure of human mitochondrial RNA polymerase. Nature.

[39] Arnold J.J., Smidansky E.D., Moustafa I.M., Cameron C.E. (2012). Human mitochondrial RNA polymerase: structure-function, mechanism and inhibition. Biochim. Biophys. Acta.

[40] Barshad G., Marom S., Cohen T., Mishmar D. (2018). Mitochondrial DNA transcription and its regulation: an evolutionary perspective. Trends Genet..

[41] Liere K., Weihe A., Börner T. (2011). The transcription machineries of plant mitochondria and chloroplasts: composition, function, and regulation. J. Plant Physiol..

[42] Černý J., Černá Bolfíková B., de A Zanotto P.M., Grubhoffer L., Růžek D. (2015). A deep phylogeny of viral and cellular right-hand polymerases. Infect. Genet. Evol..

[43] Lesburg C.A., Cable M.B., Ferrari E., Hong Z., Mannarino A.F., Weber P.C. (1999). Crystal structure of the RNA-dependent RNA polymerase from hepatitis C virus reveals a fully encircled active site. Nat. Struct. Biol..

[44] Butcher S.J., Grimes J.M., Makeyev E.V., Bamford D.H., Stuart D.I. (2001). A mechanism for initiating RNA-dependent RNA polymerization. Nature.

[45] Gong P., Peersen O.B. (2010). Structural basis for active site closure by the poliovirus RNA-dependent RNA polymerase. Proc. Natl. Acad. Sci. U. S. A..

[46] Appleby T.C., Perry J.K., Murakami E., Barauskas O., Feng J., Cho A. (2015). Viral replication. Structural basis for RNA replication by the hepatitis C virus polymerase. Science.

[47] Gong P., Kortus M.G., Nix J.C., Davis R.E., Peersen O.B. (2013). Structures of coxsackievirus, rhinovirus, and poliovirus polymerase elongation complexes solved by engineering RNA mediated crystal contacts. PLoS One.

[48] Malone B.F., Perry J.K., Olinares P.D.B., Lee H.W., Chen J., Appleby T.C. (2023). Structural basis for substrate selection by the SARS-CoV-2 replicase. Nature.

[49] Rosenqvist P., Mäkinen J.J., Palmu K., Jokinen J., Prajapati R.K., Korhonen H.J. (2022). The role of the maleimide ring system on the structure-activity relationship of showdomycin. Eur. J. Med. Chem..

[50] Malinen A.M., Turtola M., Parthiban M., Vainonen L., Johnson M.S., Belogurov G.A. (2012). Active site opening and closure control translocation of multisubunit RNA polymerase. Nucleic Acids Res..

[51] Arnold J.J., Cameron C.E. (2004). Poliovirus RNA-dependent RNA polymerase (3Dpol): pre-steady-state kinetic analysis of ribonucleotide incorporation in the presence of Mg2+. Biochemistry.

[52] Sultana S., Solotchi M., Ramachandran A., Patel S.S. (2017). Transcriptional fidelities of human mitochondrial POLRMT, yeast mitochondrial Rpo41, and phage T7 single-subunit RNA polymerases. J. Biol. Chem..

[53] Wang W., Hellinga H.W., Beese L.S. (2011). Structural evidence for the rare tautomer hypothesis of spontaneous mutagenesis. Proc. Natl. Acad. Sci. U. S. A..

[54] Bebenek K., Pedersen L.C., Kunkel T.A. (2011). Replication infidelity via a mismatch with Watson-Crick geometry. Proc. Natl. Acad. Sci. U. S. A..

[55] Batra V.K., Beard W.A., Pedersen L.C., Wilson S.H. (2016). Structures of DNA polymerase mispaired DNA termini transitioning to pre-catalytic complexes support an induced-fit fidelity mechanism. Structure.

[56] Kimsey I.J., Szymanski E.S., Zahurancik W.J., Shakya A., Xue Y., Chu C.-C. (2018). Dynamic basis for dG•dT misincorporation via tautomerization and ionization. Nature.

[57] Koag M.-C., Jung H., Lee S. (2020). Mutagenesis mechanism of the major oxidative adenine lesion 7,8-dihydro-8-oxoadenine. Nucleic Acids Res..

[58] Ferrer-Orta C., Arias A., Pérez-Luque R., Escarmís C., Domingo E., Verdaguer N. (2007). Sequential structures provide insights into the fidelity of RNA replication. Proc. Natl. Acad. Sci. U. S. A..

[59] Vassylyev D.G., Vassylyeva M.N., Zhang J., Palangat M., Artsimovitch I., Landick R. (2007). Structural basis for substrate loading in bacterial RNA polymerase. Nature.

[60] Wang D., Bushnell D.A., Westover K.D., Kaplan C.D., Kornberg R.D. (2006). Structural basis of transcription: role of the trigger loop in substrate specificity and catalysis. Cell..

[61] Cheetham G.M., Steitz T.A. (1999). Structure of a transcribing T7 RNA polymerase initiation complex. Science.

[62] Schwinghammer K., Cheung A.C.M., Morozov Y.I., Agaronyan K., Temiakov D., Cramer P. (2013). Structure of human mitochondrial RNA polymerase elongation complex. Nat. Struct. Mol. Biol..

[63] Gleghorn M.L., Davydova E.K., Basu R., Rothman-Denes L.B., Murakami K.S. (2011). X-ray crystal structures elucidate the nucleotidyl transfer reaction of transcript initiation using two nucleotides. Proc. Natl. Acad. Sci. U. S. A..

[64] Campagnola G., McDonald S., Beaucourt S., Vignuzzi M., Peersen O.B. (2015). Structure-function relationships underlying the replication fidelity of viral RNA-dependent RNA polymerases. J. Virol..

[65] Hillen H.S., Parshin A.V., Agaronyan K., Morozov Y.I., Graber J.J., Chernev A. (2017). Mechanism of transcription anti-termination in human mitochondria. Cell.

[66] Yin Y.W., Steitz T.A. (2004). The structural mechanism of translocation and helicase activity in T7 RNA polymerase. Cell.

[67] Li Y., Korolev S., Waksman G. (1998). Crystal structures of open and closed forms of binary and ternary complexes of the large fragment of Thermus aquaticus DNA polymerase I: structural basis for nucleotide incorporation. EMBO J..

[68] Zamyatkin D.F., Parra F., Alonso J.M.M., Harki D.A., Peterson B.R., Grochulski P. (2008). Structural insights into mechanisms of catalysis and inhibition in Norwalk virus polymerase. J. Biol. Chem..

[69] Yuzenkova Y., Bochkareva A., Tadigotla V.R., Roghanian M., Zorov S., Severinov K. (2010). Stepwise mechanism for transcription fidelity. BMC Biol..

[70] Chung C., Verheijen B.M., Navapanich Z., McGann E.G., Shemtov S., Lai G.-J. (2023). Evolutionary conservation of the fidelity of transcription. Nat. Commun..

[71] Xu L., Butler K.V., Chong J., Wengel J., Kool E.T., Wang D. (2014). Dissecting the chemical interactions and substrate structural signatures governing RNA polymerase II trigger loop closure by synthetic nucleic acid analogues. Nucleic Acids Res..

[72] Oh J., Kimoto M., Xu H., Chong J., Hirao I., Wang D. (2023). Structural basis of transcription recognition of a hydrophobic unnatural base pair by T7 RNA polymerase. Nat. Commun..

[73] Johnson S.J., Beese L.S. (2004). Structures of mismatch replication errors observed in a DNA polymerase. Cell.

[74] Zhang J., Palangat M., Landick R. (2010). Role of the RNA polymerase trigger loop in catalysis and pausing. Nat. Struct. Mol. Biol..

[75] Kaplan C.D., Larsson K.-M., Kornberg R.D. (2008). The RNA polymerase II trigger loop functions in substrate selection and is directly targeted by alpha-amanitin. Mol. Cell.

[76] Kireeva M.L., Nedialkov Y.A., Cremona G.H., Purtov Y.A., Lubkowska L., Malagon F. (2008). Transient reversal of RNA polymerase II active site closing controls fidelity of transcription elongation. Mol. Cell.

[77] Nedialkov Y.A., Opron K., Assaf F., Artsimovitch I., Kireeva M.L., Kashlev M. (2013). The RNA polymerase bridge helix YFI motif in catalysis, fidelity and translocation. Biochim. Biophys. Acta.

[78] Larson M.H., Zhou J., Kaplan C.D., Palangat M., Kornberg R.D., Landick R. (2012). Trigger loop dynamics mediate the balance between the transcriptional fidelity and speed of RNA polymerase II. Proc. Natl. Acad. Sci. U. S. A..

[79] Komissarova N., Kashlev M. (1997). Transcriptional arrest: Escherichia coli RNA polymerase translocates backward, leaving the 3’ end of the RNA intact and extruded. Proc. Natl. Acad. Sci. U. S. A..

[80] Nudler E., Mustaev A., Lukhtanov E., Goldfarb A. (1997). The RNA-DNA hybrid maintains the register of transcription by preventing backtracking of RNA polymerase. Cell.

[81] Wang D., Bushnell D.A., Huang X., Westover K.D., Levitt M., Kornberg R.D. (2009). Structural basis of transcription: backtracked RNA polymerase II at 3.4 angstrom resolution. Science.

[82] Cheung A.C.M., Cramer P. (2011). Structural basis of RNA polymerase II backtracking, arrest and reactivation. Nature.

[83] Abdelkareem M., Saint-André C., Takacs M., Papai G., Crucifix C., Guo X. (2019). Structural basis of transcription: RNA polymerase backtracking and its reactivation. Mol. Cell..

[84] Sekine S., Murayama Y., Svetlov V., Nudler E., Yokoyama S. (2015). The ratcheted and ratchetable structural states of RNA polymerase underlie multiple transcriptional functions. Mol. Cell.

[85] Zenkin N., Yuzenkova Y., Severinov K. (2006). Transcript-assisted transcriptional proofreading. Science.

[86] Sydow J.F., Brueckner F., Cheung A.C.M., Damsma G.E., Dengl S., Lehmann E. (2009). Structural basis of transcription: mismatch-specific fidelity mechanisms and paused RNA polymerase II with frayed RNA. Mol. Cell.

[87] Mishanina T.V., Palo M.Z., Nayak D., Mooney R.A., Landick R. (2017). Trigger loop of RNA polymerase is a positional, not acid-base, catalyst for both transcription and proofreading. Proc. Natl. Acad. Sci. U. S. A..

[88] Erie D.A., Hajiseyedjavadi O., Young M.C., von Hippel P.H. (1993). Multiple RNA polymerase conformations and GreA: control of the fidelity of transcription. Science.

[89] Reines D. (1992). Elongation factor-dependent transcript shortening by template-engaged RNA polymerase II. J. Biol. Chem..

[90] Borukhov S., Sagitov V., Goldfarb A. (1993). Transcript cleavage factors from E. coli. Cell.

[91] Izban M.G., Luse D.S. (1992). The RNA polymerase II ternary complex cleaves the nascent transcript in a 3’----5’ direction in the presence of elongation factor SII. Genes Dev..

[92] Ruan W., Lehmann E., Thomm M., Kostrewa D., Cramer P. (2011). Evolution of two modes of intrinsic RNA polymerase transcript cleavage. J. Biol. Chem..

[93] Kang J.Y., Mishanina T.V., Bao Y., Chen J., Llewellyn E., Liu J. (2023). An ensemble of interconverting conformations of the elemental paused transcription complex creates regulatory options. Proc. Natl. Acad. Sci. U. S. A..

[94] Zuber P.K., Said N., Hilal T., Wang B., Loll B., González-Higueras J. (2024). Concerted transformation of a hyper-paused transcription complex and its reinforcing protein. Nat. Commun..

[95] Skinner A., Yang C.-H., Hincks K., Wang H., Resendiz M.J.E. (2020). Experimental and theoretical rationalization for the base pairing abilities of inosine, guanosine, adenosine, and their corresponding 8-oxo-7,8-dihydropurine, and 8-bromopurine analogues within A-form duplexes of RNA. Biopolymers.

[96] Chim N., Jackson L.N., Trinh A.M., Chaput J.C. (2018). Crystal structures of DNA polymerase I capture novel intermediates in the DNA synthesis pathway. eLife.

[97] Wu E.Y., Beese L.S. (2011). The structure of a high fidelity DNA polymerase bound to a mismatched nucleotide reveals an “ajar” intermediate conformation in the nucleotide selection mechanism. J. Biol. Chem..

[98] Temiakov D., Patlan V., Anikin M., McAllister W.T., Yokoyama S., Vassylyev D.G. (2004). Structural basis for substrate selection by t7 RNA polymerase. Cell.

[99] Seley-Radtke K.L., Yates M.K. (2018). The evolution of nucleoside analogue antivirals: a review for chemists and non-chemists. Part 1: early structural modifications to the nucleoside scaffold. Antivir. Res..

[100] Yates M.K., Seley-Radtke K.L. (2019). The evolution of antiviral nucleoside analogues: a review for chemists and non-chemists. Part II: complex modifications to the nucleoside scaffold. Antivir. Res..

[101] Huang Y., Eckstein F., Padilla R., Sousa R. (1997). Mechanism of ribose 2’-group discrimination by an RNA polymerase. Biochemistry.

[102] Mäkinen J.J., Shin Y., Vieras E., Virta P., Metsä-Ketelä M., Murakami K.S. (2021). The mechanism of the nucleo-sugar selection by multi-subunit RNA polymerases. Nat. Commun..

[103] Gohara D.W., Arnold J.J., Cameron C.E. (2004). Poliovirus RNA-dependent RNA polymerase (3Dpol): kinetic, thermodynamic, and structural analysis of ribonucleotide selection. Biochemistry.

[104] Arnold J.J., Sharma S.D., Feng J.Y., Ray A.S., Smidansky E.D., Kireeva M.L. (2012). Sensitivity of mitochondrial transcription and resistance of RNA polymerase II dependent nuclear transcription to antiviral ribonucleosides. PLoS Pathog..

[105] Shannon A., Fattorini V., Sama B., Selisko B., Feracci M., Falcou C. (2022). A dual mechanism of action of AT-527 against SARS-CoV-2 polymerase. Nat. Commun..

[106] Henderson J.F., Paterson A.R., Caldwell I.C., Hori M. (1967). Biochemical effects of formycin, an adenosine analog. Cancer Res..

[107] Hori M., Takita T., Koyama G., Tadeuchi T., Umezawa H. (1964). A new antibiotic, formycin. J. Antibiot..

[108] Takeuchi T., Iwanaga J., Aoyagi T., Umezawa H. (1966). Antiviral effect of formycin and formycin B. J. Antibiot..

[109] Ishida N., Homma M., Kumagai K., Shimizu Y., Matsumoto S. (1967). Studies on the antiviral activity of formycin. J. Antibiot..

[110] Dangerfield T.L., Huang N.Z., Johnson K.A. (2020). Remdesivir is effective in combating COVID-19 because it is a better substrate than ATP for the viral RNA-dependent RNA polymerase. iScience.

[111] Seifert M., Bera S.C., van Nies P., Kirchdoerfer R.N., Shannon A., Le T.-T.-N. (2021). Inhibition of SARS-CoV-2 polymerase by nucleotide analogs from a single-molecule perspective. eLife.

[112] Svetlov V., Artsimovitch I. (2015). Purification of bacterial RNA polymerase: tools and protocols. Methods Mol. Biol..

[113] Furman R., Tsodikov O.V., Wolf Y.I., Artsimovitch I. (2013). An insertion in the catalytic trigger loop gates the secondary channel of RNA polymerase. J. Mol. Biol..

[114] Guillerez J., Lopez P.J., Proux F., Launay H., Dreyfus M. (2005). A mutation in T7 RNA polymerase that facilitates promoter clearance. Proc. Natl. Acad. Sci. U. S. A..

[115] Komissarova N., Kireeva M.L., Becker J., Sidorenkov I., Kashlev M. (2003). Engineering of elongation complexes of bacterial and yeast RNA polymerases. Meth. Enzymol..

[116] Abramoff M.D., Magalhaes P.J., Ram S.J. (2004). Image processing with ImageJ. Biophotonics Int..

[117] Nudler E., Avetissova E., Markovtsov V., Goldfarb A. (1996). Transcription processivity: protein-DNA interactions holding together the elongation complex. Science.

[118] Kandel E.S., Nudler E. (2002). Template switching by RNA polymerase II in vivo. Evidence and implications from a retroviral system. Mol. Cell.

[119] Gholamalipour Y., Karunanayake Mudiyanselage A., Martin C.T. (2018). 3’ end additions by T7 RNA polymerase are RNA self-templated, distributive and diverse in character-RNA-Seq analyses. Nucleic Acids Res..

[120] Johnson K.A. (2009). Fitting enzyme kinetic data with KinTek global kinetic explorer. Meth. Enzymol..

[121] Belogurov G.A., Vassylyeva M.N., Svetlov V., Klyuyev S., Grishin N.V., Vassylyev D.G. (2007). Structural basis for converting a general transcription factor into an operon-specific virulence regulator. Mol. Cell.

[122] Heikinheimo P., Pohjanjoki P., Helminen A., Tasanen M., Cooperman B.S., Goldman A. (1996). A site-directed mutagenesis study of Saccharomyces cerevisiae pyrophosphatase. Functional conservation of the active site of soluble inorganic pyrophosphatases. Eur. J. Biochem..

