# Supplementary material for: Probing the nucleobase selectivity of RNA polymerases with dual-coding substrates
Source: J Biol Chem. 2024 Sep 12;300(10):107755. doi: 10.1016/j.jbc.2024.107755 (PMC11474200; doi:10.1016/j.jbc.2024.107755)
Supplement: Supporting information [file mmc1.pdf]

Supplementary information

Probing the nucleobase selectivity of RNA polymerases with dual-coding substrates

Janne J. Mäkinen, Petja Rosenqvist, Pasi Virta, Mikko Metsä-Ketelä, Georgiy A. Belogurov

Supplementary Table S1. DNA oligonucleotides.

Name	Type	Sequence (5’ to 3’)
S001	tDNA	GTCTCATCTGGCATTGTACCTCCTCTTAAACCTTAGATCGCTACAGTC
S028	ntDNA	GACTGTAGCGATCTAAGGTTTAAGAGGAGGTACAATGCCAGATGAGAC
S046	tDNA	GCTACTCTACTGACATGACCGCTCCTCTGGAACCTTAGATCGCTACAAGT
S049	ntDNA	ACTTGTAGCGATCTAAGGTTCCAGAGGAGCGGTCATGTCAGTAGAGTAGC
S272	tDNA	CGTCTCATCTGGCATCATCGCTCCTCTTAAACCTTAGATCGCTACAGTCG
S273	ntDNA	CGACTGTAGCGATCTAAGGTTTAAGAGGAGCGATGATGCCAGATGAGACG
S274	tDNA	GCTCTCATCTGGACATGTGCACTCCTCTTAAACCTTAGATCGCTACAGTC
S275	ntDNA	GACTGTAGCGATCTAAGGTTTAAGAGGAGTGCACATGTCCAGATGAGAGC
S276	tDNA	GCCTTCATCTGCACAGCACGTCTCCTCTTAAACCTTAGATCGCTACAGTC
S277	ntDNA	GACTGTAGCGATCTAAGGTTTAAGAGGAGACGTGCTGTGCAGATGAAGGC
S308M	tDNA	GCTACTCTACTGCACTATXGTCTCCTCTTAACTCTTAGATCGCTACAAGT
S309	ntDNA	ACTTGTAGCGATCTAAGAGTTAAGAGGAGACCATAGTGCAGTAGAGTAGC
S310M	tDNA	GCTACTCTACGTCACTATXCGCTCCTCTTAAACCTTAGATCACTACAAGT
S311	ntDNA	ACTTGTAGTGATCTAAGGTTTAAGAGGAGCGCATAGTGACGTAGAGTAGC
S321	tDNA	GTACTGTTACTGATACTAGTGTACGCATGCGAGTCTAATCTGTTCTGCTCTCCTCTTAAACCTTACACTG
S322	ntDNA	CAGTGTAAGGTTTAAGAGGAGAGCAGAACAGATTAGACTCGCATGCGTACACTAGTATCAGTAACAGTAC
S353	tDNA	CGCTATAGTCGCTCTCATCTGTCAAAAAAAACTCCTCTTAAACCTTAGAT
S354	ntDNA	ATCTAAGGTTTAAGAGGAGTTTTTTTTGACAGATGAGAGCGACTATAGCG
S363	tDNA	GCCTTCATCTGCACAATGCATCTCCTCTTAAACCTTAGATCGCTACAGTC
S364	ntDNA	GACTGTAGCGATCTAAGGTTTAAGAGGAGATGCATTGTGCAGATGAAGGC
S365	tDNA	GTACTGTTACTGATACTAGTGTACGCATGCGAGTCTAATGAGTACTGAGCTCCTCTTAAACCTTACACTG
S366	ntDNA	CAGTGTAAGGTTTAAGAGGAGCTCAGTACTCATTAGACTCGCATGCGTACACTAGTATCAGTAACAGTAC
S373	tDNA	ACACACTAATACGACTCACTATAGGTACTGTTACTGATACTAGCGTACACGTACCAGTCTGATCTGAACGCGCCTCCTCT GGTTGTGTGTGCGT
S374	ntDNA	ACGCACACACAACCAGAGGAGGCGCGTTCAGATCAGACTGGTACGTGTACGCTAGTATCAGTAACAGTACCTATAGTGAG TCGTATTAGTGTGT
S385	tDNA	GTACTGATACTGATACAGCTATACGCATGCTAGTCTAGTCTATTCTACTCTCCTCTTAAACCTTACACTG
S386	ntDNA	CAGTGTAAGGTTTAAGAGGAGAGTAGAATAGACTAGACTAGCATGCGTATAGCTGTATCAGTATCAGTAC
S389	tDNA	GTACTGTTACGTATACATGTGCTAGCAGTCAAGTCTACTATGTTATGATCTCCTCTTAAACCTTACACTG
S390	ntDNA	CAGTGTAAGGTTTAAGAGGAGATCATAACATAGTAGACTTGACTGCTAGCACATGTATACGTAACAGTAC
S393	tDNA	ACACACTAATACGACTCACTATAGGTACTGTTACTGATACTAGCGTACACGTACTCATCTGATCTGAACGCGCCTCCTCT GGTTGTGTGTGCGT
S394	ntDNA	ACGCACACACAACCAGAGGAGGCGCGTTCAGATCAGATGAGTACGTGTACGCTAGTATCAGTAACAGTACCTATAGTGAG TCGTATTAGTGTGT
S399	tDNA	CGTCTCATCTGGCATCATCICTCCTCTTAAACCTTAGATCGCTACAGTCG

Color coding: 2’-deoxyribonucleotides black. X=6-methyl-isoxanthopterin. I=inosine

Supplementary Table S2. RNA oligonucleotides.

Name	Type	Sequence (5' to 3')
R002	RNA	Atto680-CACUAACUAAGAGGAG
R047	RNA	Atto680-CUCACAUCCAGAGGAG
R098	RNA	Atto680-CACACGCACACACAACCAGAGGAG
R131	RNA	CACGACUGAUGCCUCCUCUUAACA
R132	RNA	CAUCACUGAUCGCUCCUCUUAACA
R133	RNA	ACCAGCUAACGUCUCCUCUUAACA
R134	RNA	ACUGACUAUGCACUCCUCUUAACA
RS373	RNA	GGUACUGUUACUGAUACUAGCGUACACGUACCAGUCUGAUCUGAACGCGCCUCCUCUGGUUGUGUGUGCGU
RS393	RNA	GGUACUGUUACUGAUACUAGCGUACACGUACUCAUCUGAUCUGAACGCGCCUCCUCUGGUUGUGUGUGCGU
RS393F	RNA	GGUFCUGUUFCUGFUFCUFGCGUFCFCGUFCUCFUCUGFUCUGFFCGCGCCUCCUCUGGUUGUGUGUGCGU

Color coding: ribonucleotides red, 2'OMe-2'-deoxyribonucleotides blue. F=FOR

Supplementary Table S3. Protein expression vectors.

Name	Description	Source/reference
pVS10	Ec-DdRP (T7p-α-β-β'_His$_6$-ω)	Belogurov *et al*, 2007
pRP009	Mt-DdRP (T7p-His6_TEV_RNAP)	Rosenqvist *et al*, 2022
pSP011	Δ214 Mt-DdRP[H1125A] (T7p-His6_TEV_RNAP)	this work
pGB161	Cv-RdRP (T7p-His6_TEV_RNAP)	this work
pIA578	*E. coli* GreA (T7p-GreA_His$_6$)	Furman *et al*, 2013
pBH161-P266L	T7 RNAP P266L	Guillerez et al, 2005
pYWT	*S. cerevisiae* PPase (tucP-PPase)	Heikinheimo et al, 1996

Supplementary references

Belogurov G.A., Vassylyeva M.N., Svetlov V., Klyuyev S., Grishin N.V., Vassylyev D.G. & Artsimovitch I. (2007) Structural basis for converting a general transcription factor into an operon-specific virulence regulator. *Mol Cell.* 26(1):117-29.

Guillerez J, Lopez PJ, Proux F, Launay H & Dreyfus M (2005) A mutation in T7 RNA polymerase that facilitates promoter clearance. *Proc. Natl. Acad. Sci. USA* 102: 5958–5963.

Furman R., Tsodikov O.V., Wolf Y.I. & Artsimovitch I (2013) An insertion in the catalytic trigger loop gates the secondary channel of RNA polymerase. *J. Mol. Biol.* 425: 82–93.

Heikinheimo, P., Pohjanjoki, P., Helminen, A., Tasanen, M., Cooperman, B. S., Goldman, A., Baykov, A., & Lahti, R. (1996). A site-directed mutagenesis study of *Saccharomyces cerevisiae* pyrophosphatase. Functional conservation of the active site of soluble inorganic pyrophosphatases. *Eur. J. Biochem*, 239(1), 138–143.

Rosenqvist P.; Mäkinen J. J.; Palmu K.; Jokinen J.; Prajapati R. K.; Korhonen H. J.; Virta P.; Belogurov G. A.; Metsä-Ketelä M. The role of the maleimide ring system on the structure-activity relationship of showdomycin. *Eur. J. Med. Chem.* 2022, 237, 114342.

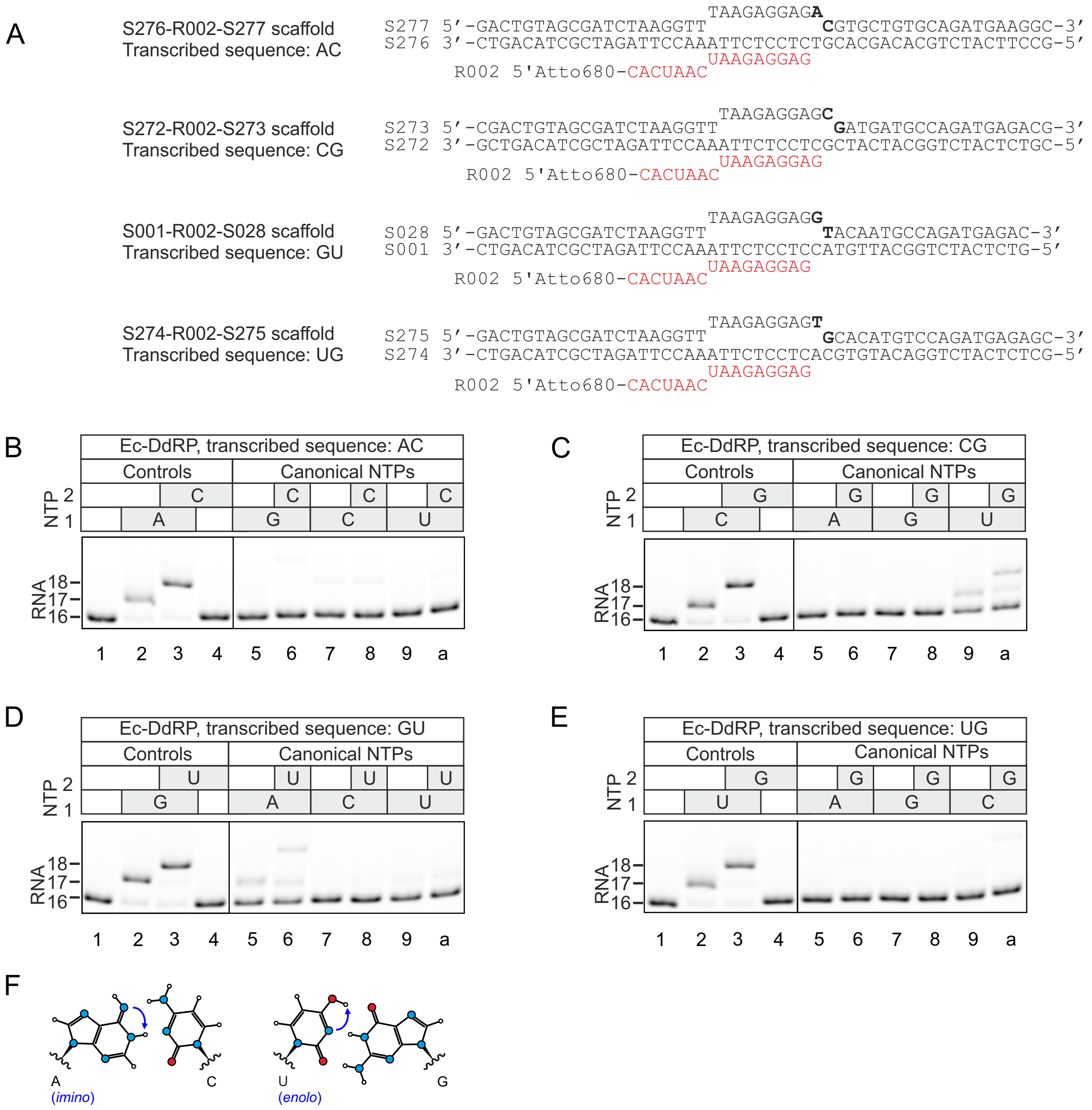

Figure S1. RNA extension with non-cognate canonical NMPs by Ec-DdRP. Cognate NTPs were added at 10 μM, non-cognate canonical NTPs were added at 100 μM. The reaction mixtures were incubated for 1 min at 25 °C. **(A)** Schematics of nucleic acid scaffolds. **(B)** Misincorporation of G, C or U in place of adenine. **(C)** Misincorporation of A, G, or U in place of cytidine. **(D)** Misincorporation of A, C, or U in place of guanine. **(E)** Misincorporation of A, G, or C in place of uridine. **(F)** Nucleobase pairings that may be responsible for nucleotide misincorporations in (C and D). A rare *imino* tautomer of substrate adenine (or acceptor cytidine, not depicted) likely accounts for misincorporation of AMP against cytidine acceptor nucleotide (D, *lanes 5-6*). A rare *enol* tautomer of substrate uridine (or acceptor guanine, not depicted) likely accounts for misincorporation of UMP against guanine acceptor nucleotide (C, *lanes 9-a*). Blue arrows signify tautomerization that likely takes place as a part of base pairing. Quantification is presented in the Supplementary Data File.

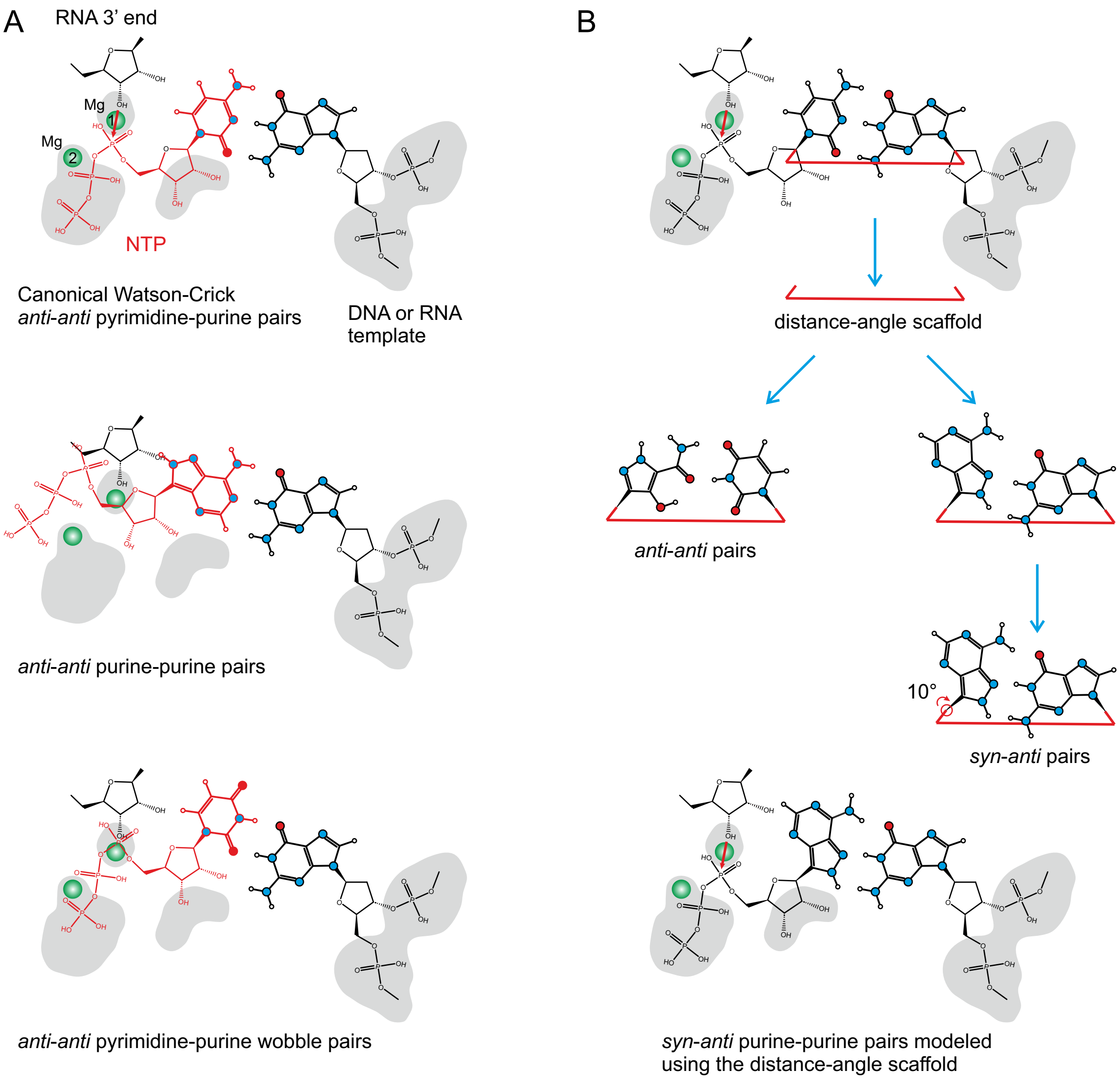

Figure S2. The productive base pairings within the RNAP active site must maintain both the inter-nucleo-sugar distance and the angle between the glycosidic bonds. (A) Substrate NTP binding involves accommodating the nucleo-sugar in a specific pocket (gray shape) and coordinating the pyrophosphate moiety in a dedicated location (gray shape) within the active site. Additionally, the positions of the acceptor nucleobase and the RNA 3’OH group, which ultimately attacks the α-phosphate of the NTP leading to nucleotide incorporation, are constrained by interactions with the protein surface (gray shapes). Purine-purine base pairs mediated by *anti* conformers of nucleotides (e.g. FOR-guanine pair, middle row illustration) or pyrimidine-purine wobble pairs (e.g. uridine-guanine pair, bottom row illustration) result in weak binding because only a subset of interactions between the NTP and the active site can be established. These pairings are also unlikely to result in nucleotide incorporation due to improper alignment of the reactants for catalysis. **(B)** Using a canonical Watson-Crick base pair, we constructed a distance-angle scaffold (red polyline) to model potentially productive base pairs that preserve the inter-nucleo-sugar distance and the angle between the glycosidic bonds. For *syn-anti* pairs, a 10° adjustment of the angle between the glycosidic bonds was necessary to achieve a plausible pairing geometry. Base pairs modeled using the distance-angle scaffold principally allow proper positioning of the nucleo-sugar and pyrophosphate moiety of the incoming NTP for binding and catalysis.

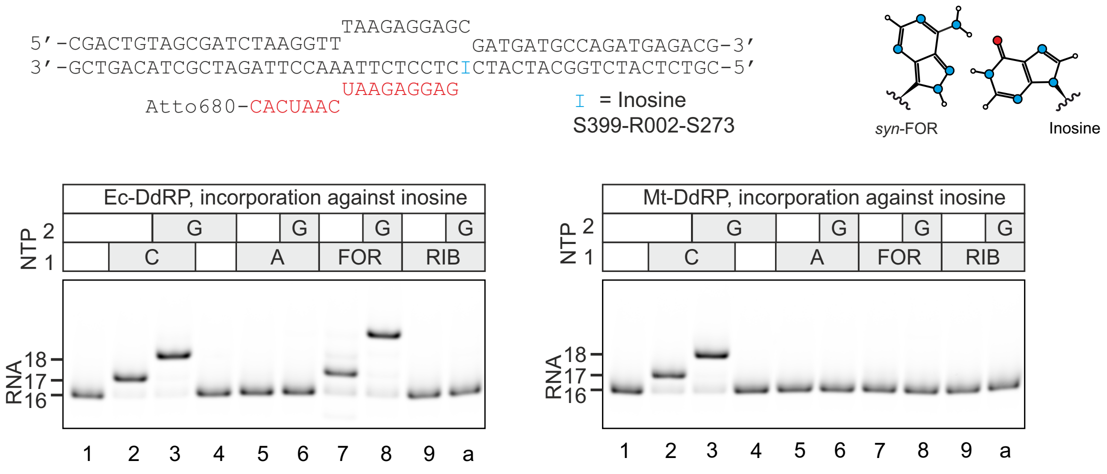

Figure S3. Incorporation of nucleoside analogues into RNA against inosine acceptor base. Assembled ECs were supplemented with 10 µM NTPs, 100 µM nucleoside analogues and incubated for 1 min at 25 °C. Scaffold schematics and *syn*-FOR pair with inosine acceptor base are shown above the gel panels. DNA, RNA and 2'OMe nucleotides are colored black, red and cyan, respectively. Quantification is presented in the Supplementary Data File.

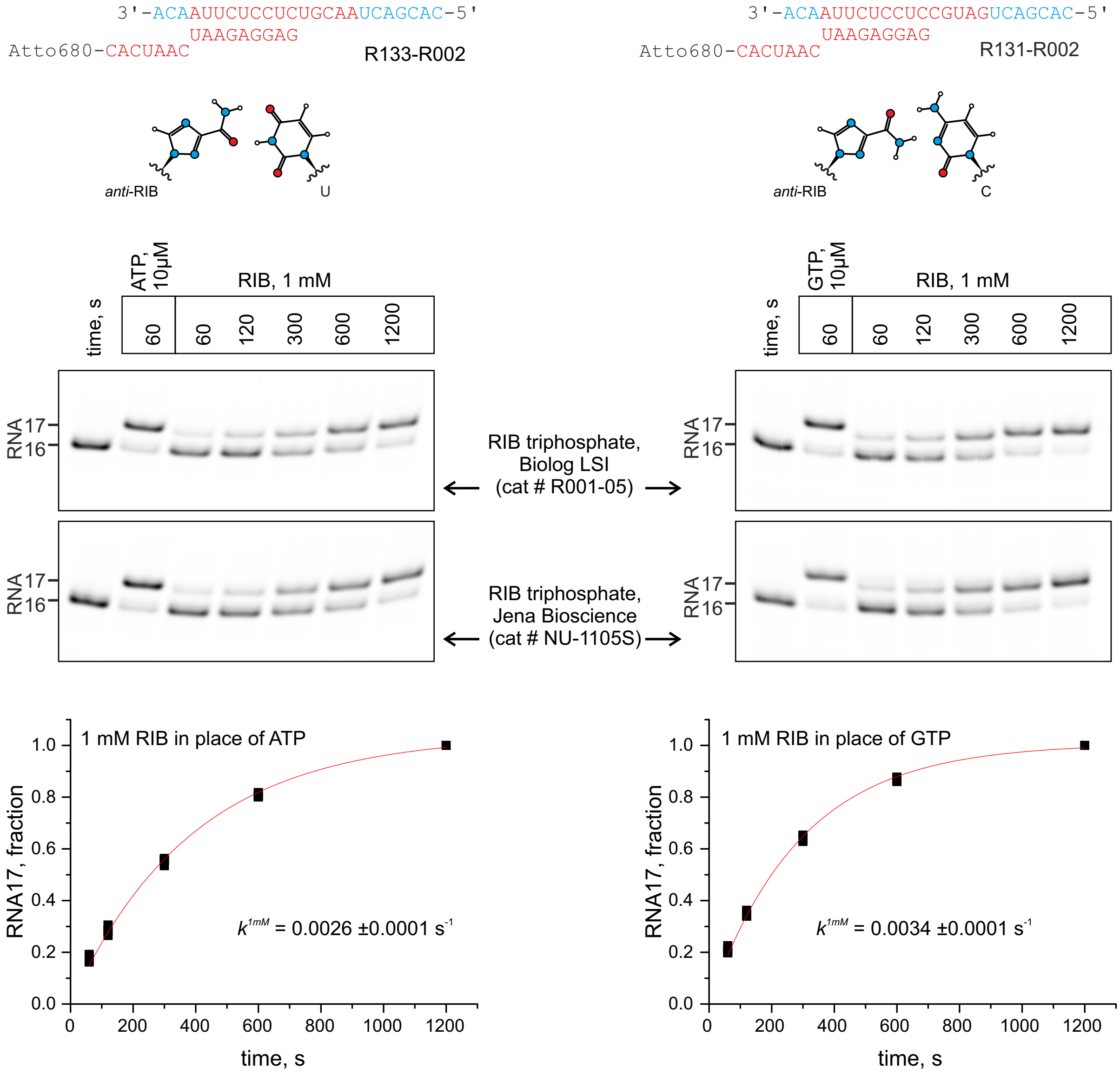

Figure S4. Cv-RdRP can incorporate RIB in place of adenine and guanine upon prolonged incubation with 1mM RIB triphosphate. Assembled ECs were supplemented with 10 µM ATP (left), GTP (right) or 1 mM RIB triphosphate and incubated for the indicated times at 25 °C. Two batches of RIB triphosphate obtained from BIOLOG Life Science Institute (top gel panels) and Jena Bioscience (bottom gel panels) were tested with similar outcome. Scaffold schematics and RIB pairs with uridine and cytidine acceptor bases are shown above the gel panels. Fractions of extended RNA (four replicates) were fit to a single exponential function (bottom graphs).

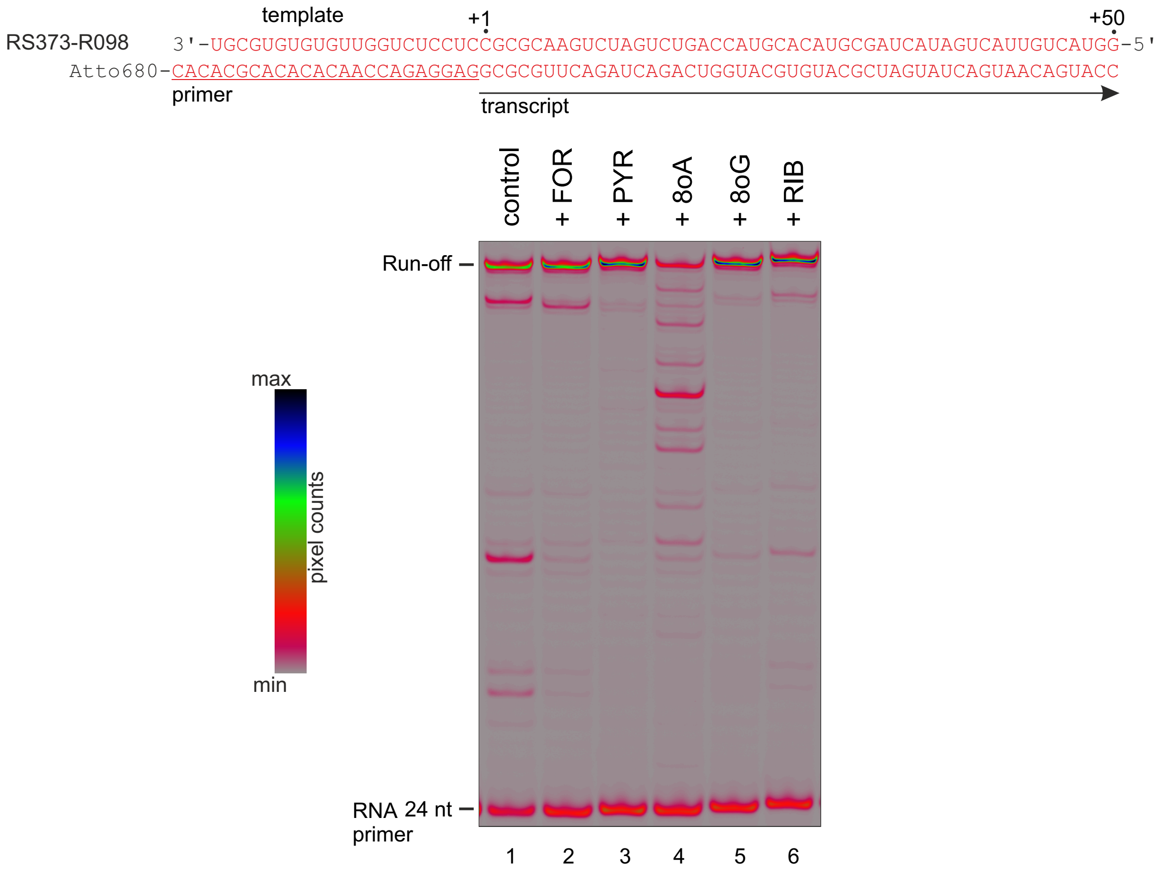

Figure S5 . The effect of triphosphorylated nucleoside analogues on processive transcription by Cv-RdRP in a competitive setup. Most experiments in this study analyzed the ability of nucleoside analogues to serve as substrates for diverse RNAPs. However, nucleoside analogues may potentially interfere with transcription without incorporating into RNA: by binding in the active site and competing with canonical NTPs or by binding elsewhere and allosterically interfering with the catalysis in the RNAP active site. Considering viral RdRPs are intended targets of inhibition by nucleoside analogue drugs, we additionally investigated the effects of nucleoside analogues on the RNA synthesis by Cv-RdRP in a competitive mode. ECs were chased with four canonical NTPs (*lane 1*), or four canonical NTPs and a 20-fold excess of triphosphorylated nucleoside analogues (*lanes* 2-7) for 5 minutes at 25 °C. NTPs and triphosphorylated nucleoside analogues were added at 100 and 2000 μM, respectively. The 16-bit grayscale scan was normalized using max pixel counts and pseudo-colored using RGB palette. Template RNA, RNA primer (underlined) and the RNA transcript synthesized from canonical NTPs are shown above the gel. RNA primer was chemically synthesized, RNA template was produced by *in vitro* transcription with T7 RNAP P266L. Most nucleoside analogue improved the transcription by diminishing pausing and arrests at several sites. The presence of high concentration of the triphosphorylated nucleoside analogue seemed to be the key for stimulation, but the exact mechanism is unclear. The only nucleoside analogue that caused a mild inhibition by inducing arrests at internal sites was 8oA triphosphate. Considering that 8oA displays a Trojan-horse-like behavior when incorporated in place of adenine by Ec-DdRP (Figure 4B *lane 6*), we speculate that a similar effect may account for its interference with the Cv-RdRP transcription. At high concentration of 2 mM employed in the competitive assay, 8oA may be incorporated in place of both adenine and uridine by Cv-RdRP and cause the inhibition of transcription downstream of the incorporation sites by distorting the structure of the nascent RNA duplex.

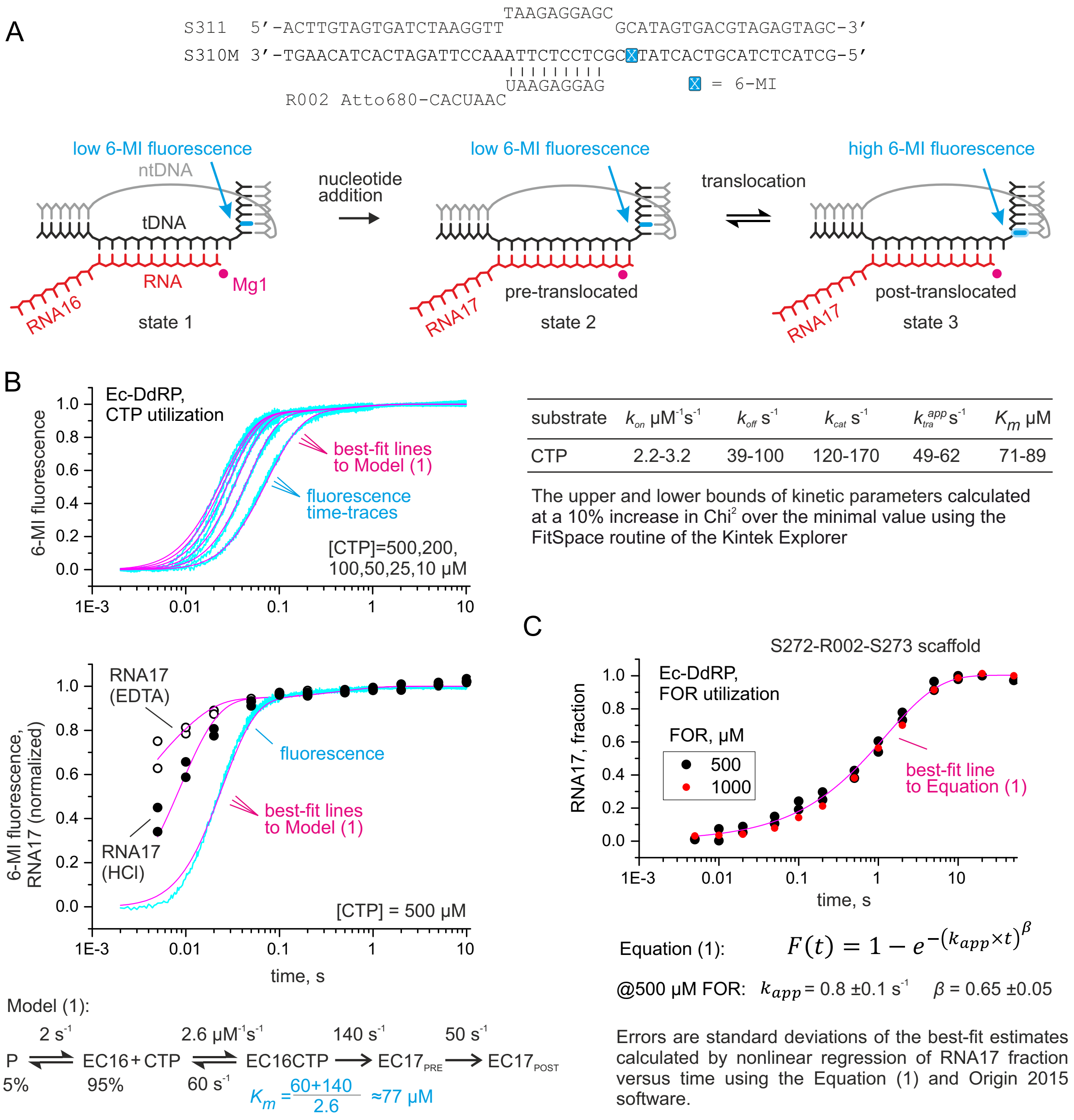

substrate	k_{on} μM^{-1}s^{-1}	k_{off} s^{-1}	k_{cat} s^{-1}	k_{tra}^{app} s^{-1}	K_m μM
CTP	2.2-3.2	39-100	120-170	49-62	71-89

Figure S6. Time-resolved measurements of FOR triphosphate and CTP utilization by Ec-DdRP. (A) The nucleic acid scaffold employed in translocation and nucleotide addition assays. The fluorescence of a guanine analogue 6-MI (cyan) was quenched by neighboring base pairs in the initial EC (state 1) and the pre-translocated EC that formed following the nucleotide incorporation (state 2) but increased when the 6-MI relocated to the edge of the downstream DNA duplex upon translocation (state 3). The template DNA, non-template DNA, RNA and the catalytic Mg^{2+} ion are colored black, gray, red and magenta, respectively. **(B)** CTP concentration series. Data were plotted and analyzed as described in Figure 8. **(C)** FOR utilization time curves obtained at 500 μM (black circles) and 1000 μM (red circles) of FOR using a quench-flow instrument and HCl as a quencher. ECs were assembled using a non-fluorescent scaffold depicted in Figure 3A. FOR utilization time curve at 500 μM was fit to Equation (1) to infer the apparent reaction rate and stretching parameter. Considering that 500 and 1000 μM time curves are largely superimposed, the apparent reaction rate is a good approximation of FOR utilization turnover.

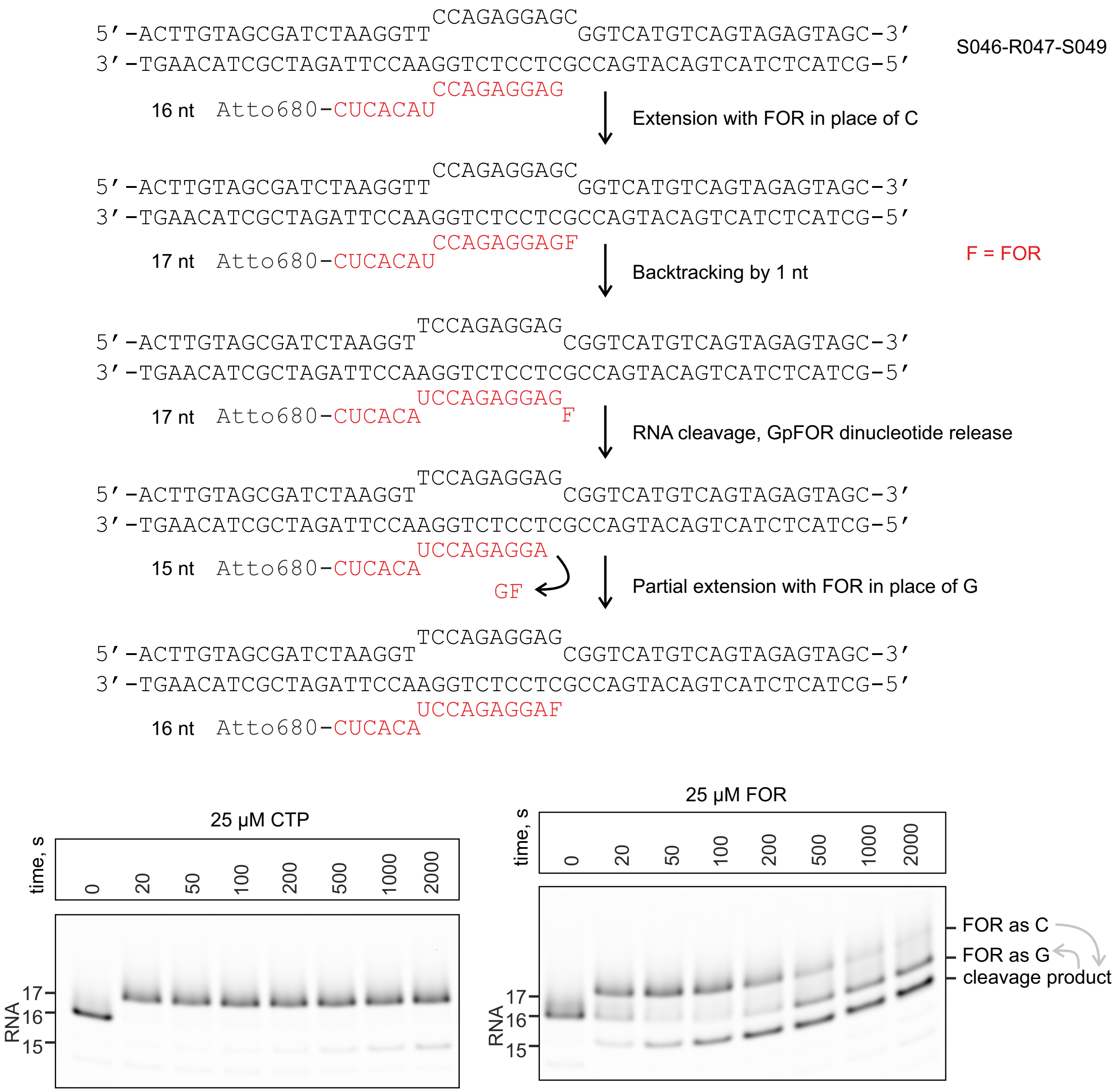

Figure S7. Incorporation of FOR in place of cytidine by Ec-DdRP triggers proofreading activity that involves backtracking and cleavage of the RNA dinucleotide. Assembled ECs were supplemented with 25 μM CTP (left panel) or FOR triphosphate (right panel) and incubated for the indicated times at 25 °C. ECs were assembled using an RNA primer with 10 bp complementarity to the template DNA, facilitating backtracking after extension by one nucleotide. In contrast, other Ec-DdRP ECs used in this study were assembled with RNA primers with 9 bp complementarity to the template DNA to disfavor backtracking. Scaffold schematics and the sequence of events during the proofreading reaction are shown above the gel panels. Incorporation of FOR instead of CMP triggers backtracking and cleavage of the RNA dinucleotide, followed by a partial misincorporation of FOR instead of GMP.

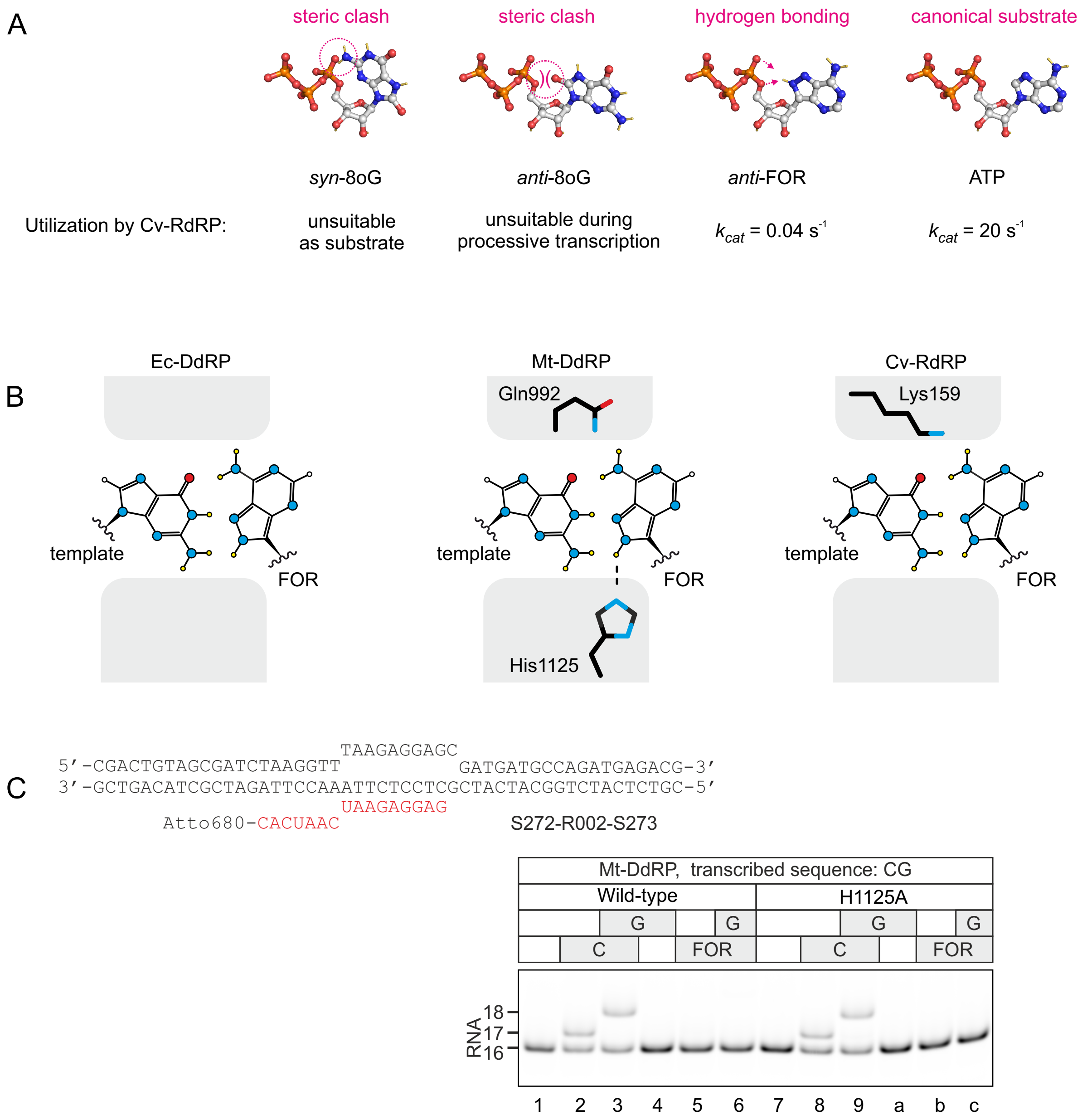

Figure S8. Cv-RdRP uniquely rejected *syn*-8oG, while Mt-DdRP uniquely rejected *syn*-FOR. (A) The inability of Cv-RdRP to efficiently utilize *syn*-8oG and several other nucleoside analogues may be due to the increased sensitivity to steric repulsion (*syn*- and *anti*-8oG) and potential attraction (*anti*-FOR) between the nucleobase and the α-phosphate of the substrate. The structures were generated by replacing the nucleobase of the UTP substrate positioned for catalysis in RdRP active site (PDB ID 7W9S) with *syn*-8oG, *anti*-8oG, *anti*-FOR and adenine. Only polar hydrogens are shown. **(B)** Right-hand but not two-barrel RNAPs feature amino acid residues capable of forming hydrogen bonds with edges of the base pair between the substrate NTP and the acceptor base. Among those, His1125 in Mt-DdRP is positioned to interact with the pyrazole moiety of *syn*-FOR and can potentially be responsible for the inability of Mt-DdRP to incorporate FOR against guanine. **(C)** H1125A substitution did not enable Mt-DdRP to incorporate *syn*-FOR against guanine, suggesting His1125 is not crucial for discrimination. Assembled ECs were supplemented with 10 μM NTPs, 100 μM FOR triphosphate and incubated for 1 min at 25 °C. Schematic of the nucleic acid scaffold is presented above the gel panel. DNA and RNA nucleotides are colored black and red, respectively. Quantification is presented in the Supplementary Data File.

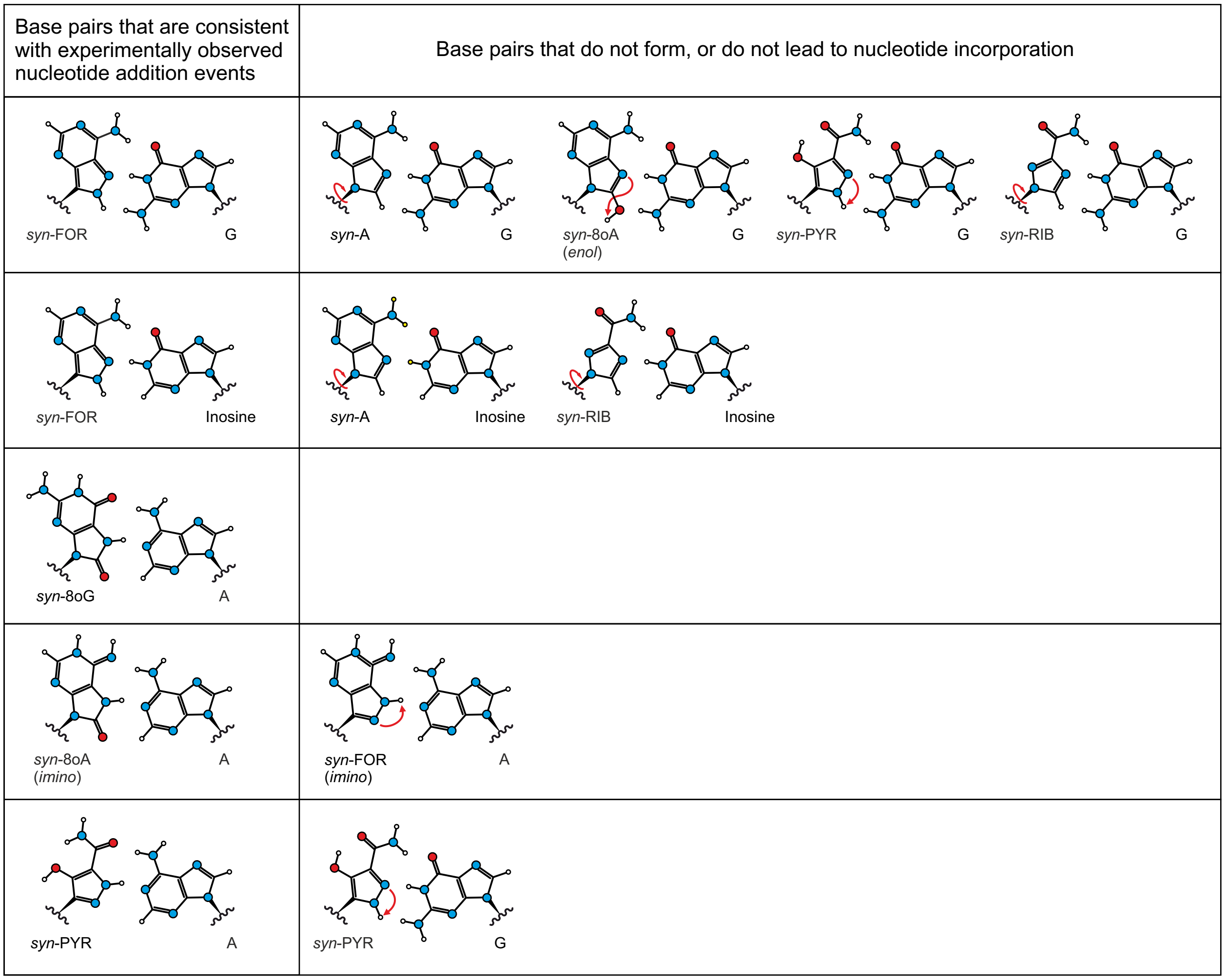

Figure S9. Only a subset of geometrically compatible *syn-anti* base pairings results in nucleotide incorporation. Red arrows signify isomerizations that do not take place or are incompatible with nucleotide incorporation. Some non-productive pairs are depicted more than once to contrast them with geometrically similar productive pairs. All base pairs were modeled as described in Figure S2.

Base pairs that are consistent with experimentally observed nucleotide addition events	Base pairs that do not form, or do not lead to nucleotide incorporation
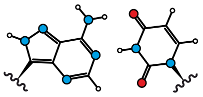 *anti*-FOR U or T	
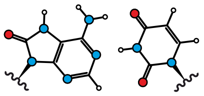 *anti*-8oA U or T	
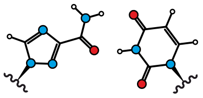 *anti*-RIB U or T	
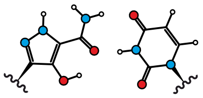 *anti*-PYR U or T	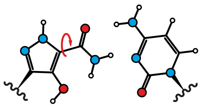 *anti*-PYR C
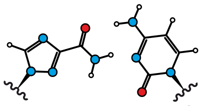 *anti*-RIB C	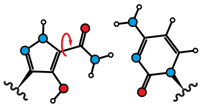 *anti*-PYR C
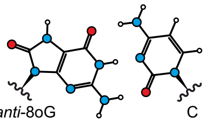 *anti*-8oG C	
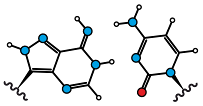 *anti*-FOR (*imino*) C	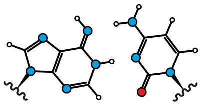 A (*imino*) C inefficient incorporation, Figure S1

Figure S10. Most geometrically compatible *anti-anti* base pairings results in nucleotide incorporation. A notable exception is *anti*-PYR pair with cytidine that does not form or does not result in PYR incorporation. Red arrows signify isomerizations that do not take place or are incompatible with nucleotide incorporation. Some non-productive pairs are depicted more than once to contrast them with geometrically similar productive pairs. All base pairs were modeled as described in Figure S2.

A

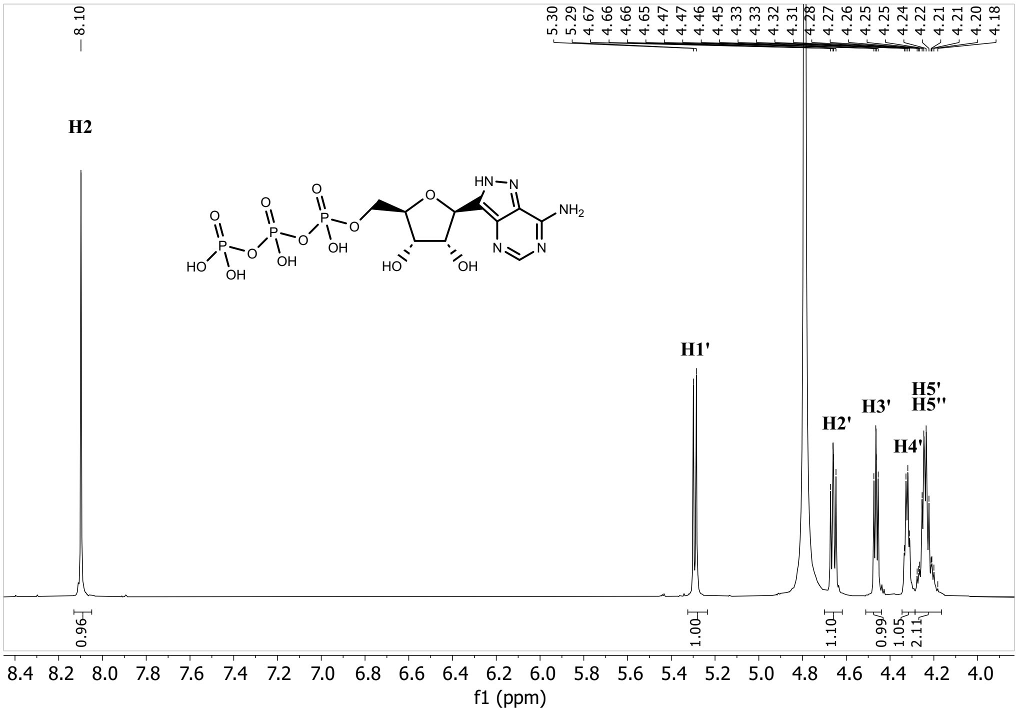

B

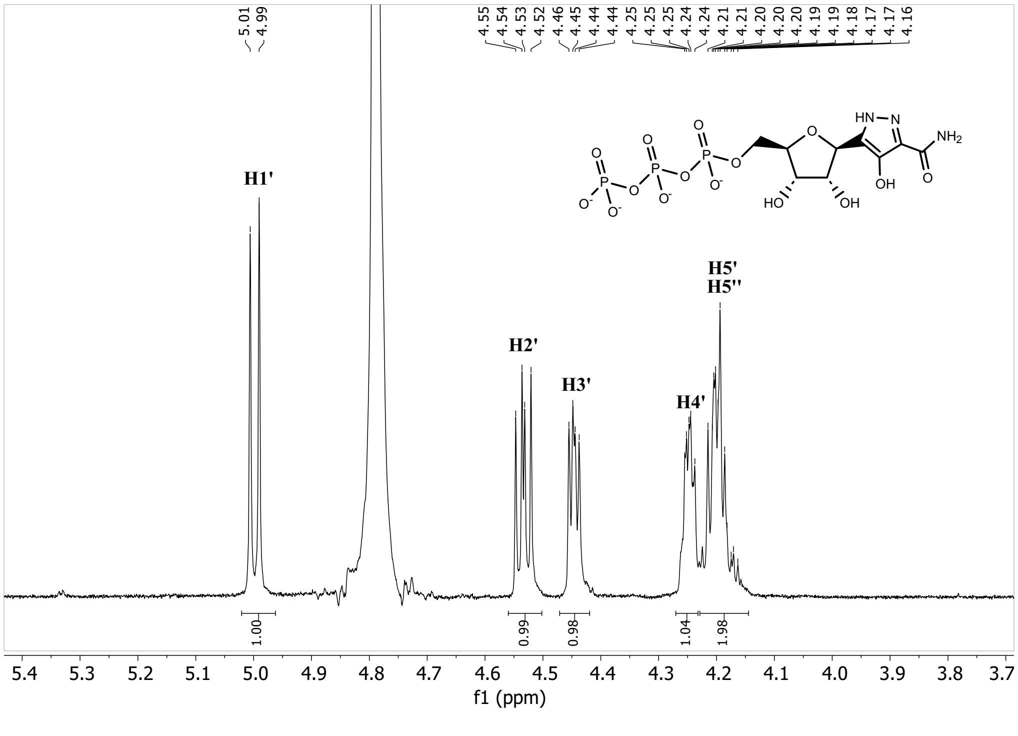

Figure S11. ^{1}H NMR (500 MHz, D_2O) spectra of FOR 5'-triphosphate **(A)** and PYR 5'-triphosphate **(B)**.

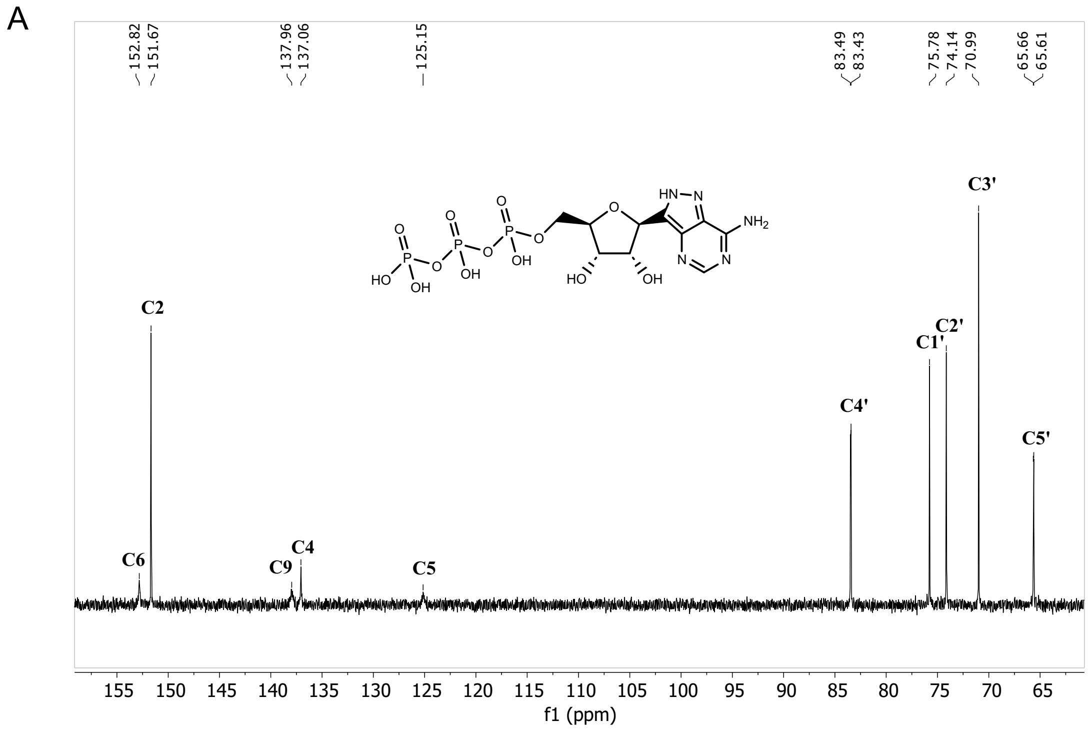

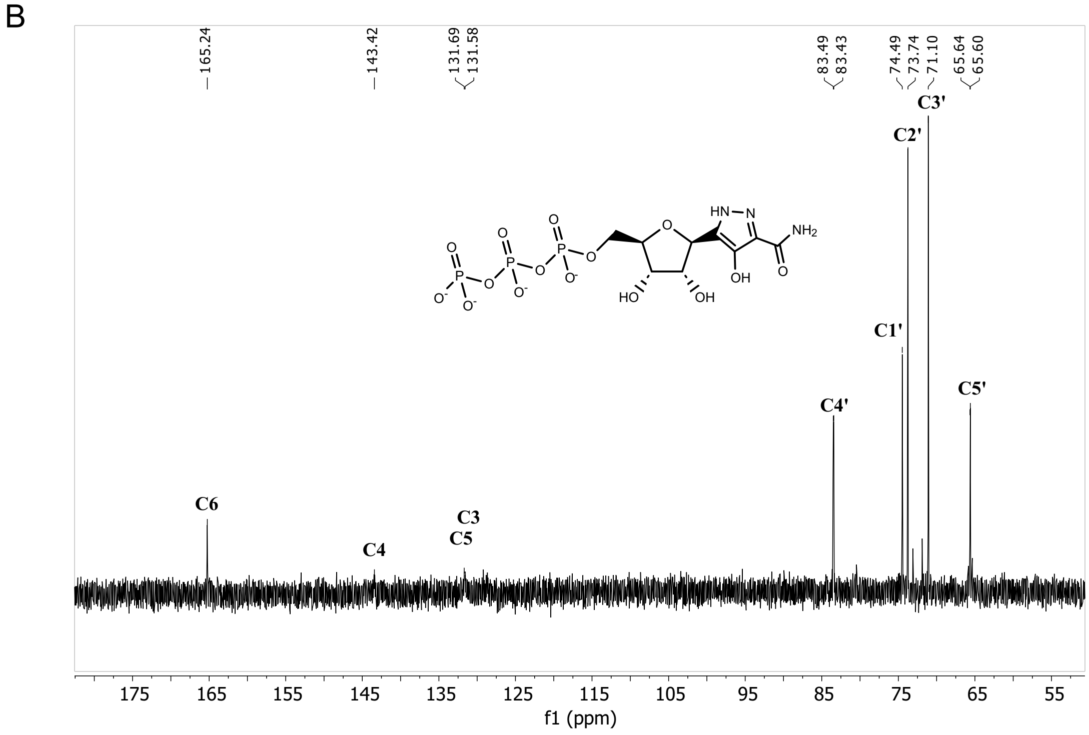

Figure S12. ^{13}C NMR (126 MHz, D_2O) spectra of FOR 5'-triphosphate **(A)** and PYR 5'-triphosphate **(B)**.

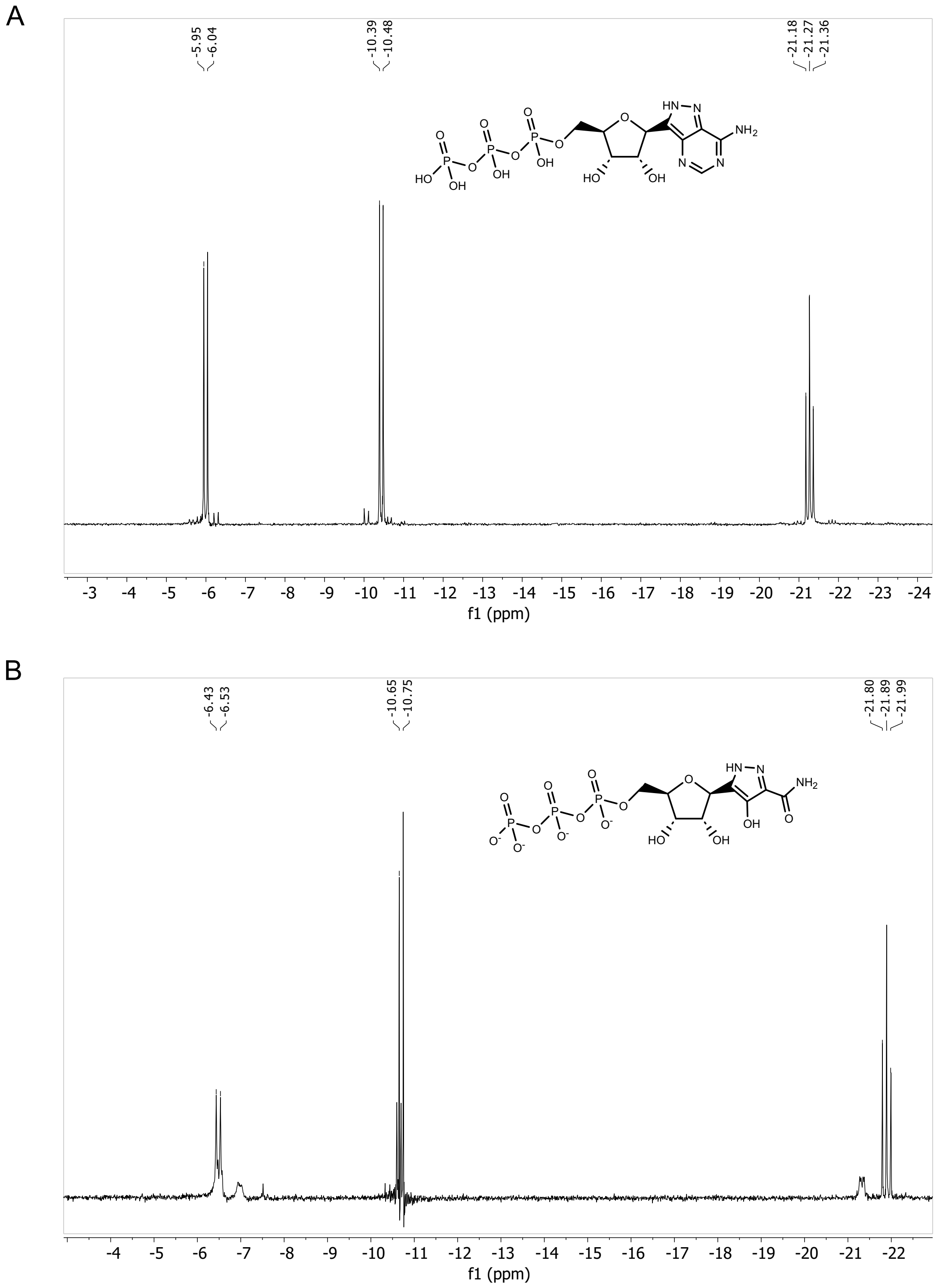

Figure S13. ^{31}P NMR (202 MHz, D_2O) spectra of FOR 5'-triphosphate **(A)** and PYR 5'-triphosphate **(B)**. The spontaneous anomerization to the inactive pyrazofurin B 5'-triphosphate (α-anomer) is evidenced by the chemical shifts appearing around -6.9, -10.6 and -21.3 ppm.

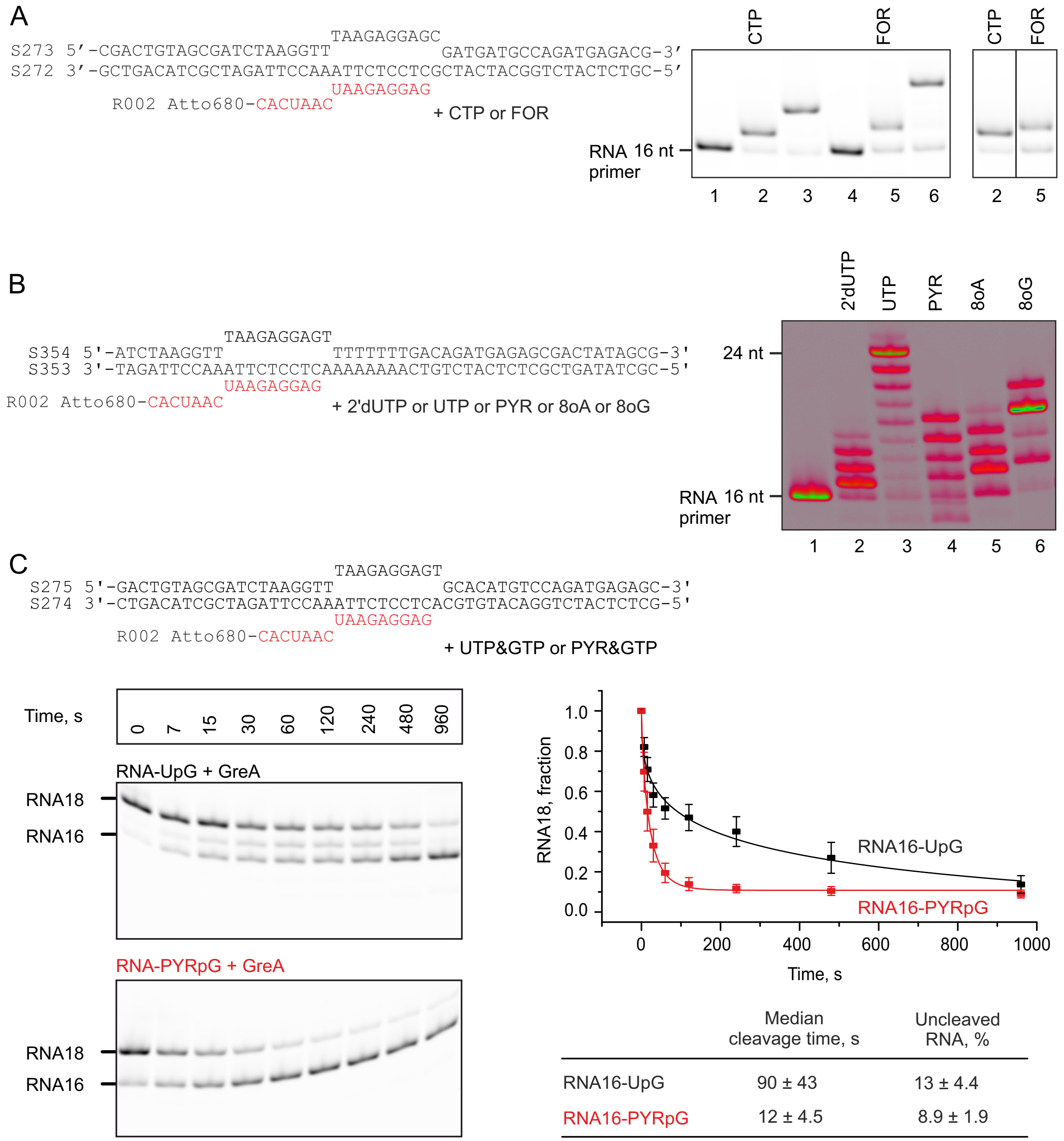

	Median cleavage time, s	Uncleaved RNA, %
RNA16-UpG	90 ± 43	13 ± 4.4
RNA16-PYRpG	12 ± 4.5	8.9 ± 1.9

Figure S14. ECs assembled with Ec-DdRP and extended with nucleoside analogues are distinct from ECs extended with canonical NMPs. **(A)** Gel is duplicated from Figure 2B. Lanes 2 and 5 are cut out and presented side by side to emphasize the difference in the electrophoretic mobility of CMP and FOR extended RNA. **(B)** Transcription of octa-uridine track in the presence 2’dUTP (100 μM), UTP (10 μM), PYR (100 μM), 8oA (100 μM) and 8oG (100 μM) for 1 h at 37°C. RNA extended with 2’dUMP (*lane 2*) migrate noticeably faster, whereas RNAs extended with 8oA (*lane 5*) and 8oG (*lane 6*) migrate noticeably slower than RNAs extended with UMP (*lane 3*). **(C)** ECs were assembled with Ec-DdRP, extended with 50 μM UTP and 50 μM GTP (top gel) or 100 μM PYR and 50 μM GTP (bottom gel), $MgCl_2$ and NTPs were removed by gel filtration. GreA assisted RNA cleavage reactions were initiated by mixing pre-extended ECs with 10 μM GreA and 10 mM $MgCl_2$. Solid lines are the best-fits to a stretched exponential function. PYRpG dinucleotide is cleaved off noticeably faster than UpG.
